# Overview of Technologies Implemented During the First Wave of the COVID-19 Pandemic: Scoping Review

**DOI:** 10.2196/29136

**Published:** 2021-09-14

**Authors:** Alaa Abd-Alrazaq, Asmaa Hassan, Israa Abuelezz, Arfan Ahmed, Mahmood Saleh Alzubaidi, Uzair Shah, Dari Alhuwail, Anna Giannicchi, Mowafa Househ

**Affiliations:** 1 Division of Information and Computing Technology, College of Science and Engineering Hamad Bin Khalifa University Qatar Foundation Doha Qatar; 2 Information Science Department Kuwait University Kuwait Kuwait; 3 Health Informatics Unit Dasman Diabetes Institute Kuwait Kuwait; 4 School of Professional Studies Berkeley College New York, NY United States

**Keywords:** technologies, digital tools, COVID-19, novel coronavirus, scoping review, digital health, telemedicine

## Abstract

**Background:**

Technologies have been extensively implemented to provide health care services for all types of clinical conditions during the COVID-19 pandemic. While several reviews have been conducted regarding technologies used during the COVID-19 pandemic, they were limited by focusing either on a specific technology (or features) or proposed rather than implemented technologies.

**Objective:**

This review aims to provide an overview of technologies, as reported in the literature, implemented during the first wave of the COVID-19 pandemic.

**Methods:**

We conducted a scoping review using PRISMA (Preferred Reporting Items for Systematic Reviews and Meta-analyses) Extension for Scoping Reviews. Studies were retrieved by searching 8 electronic databases, checking the reference lists of included studies and relevant reviews (backward reference list checking), and checking studies that cited included studies (forward reference list checking). The search terms were chosen based on the target intervention (ie, technologies) and the target disease (ie, COVID-19). We included English publications that focused on technologies or digital tools implemented during the COVID-19 pandemic to provide health-related services regardless of target health condition, user, or setting. Two reviewers independently assessed the eligibility of studies and extracted data from eligible papers. We used a narrative approach to synthesize extracted data.

**Results:**

Of 7374 retrieved papers, 126 were deemed eligible. Telemedicine was the most common type of technology (107/126, 84.9%) implemented in the first wave of the COVID-19 pandemic, and the most common mode of telemedicine was synchronous (100/108, 92.6%). The most common purpose of the technologies was providing consultation (75/126, 59.5%), followed by following up with patients (45/126, 35.7%), and monitoring their health status (22/126, 17.4%). Zoom (22/126, 17.5%) and WhatsApp (12/126, 9.5%) were the most commonly used videoconferencing and social media platforms, respectively. Both health care professionals and health consumers were the most common target users (103/126, 81.7%). The health condition most frequently targeted was COVID-19 (38/126, 30.2%), followed by any physical health conditions (21/126, 16.7%), and mental health conditions (13/126, 10.3%). Technologies were web-based in 84.1% of the studies (106/126). Technologies could be used through 11 modes, and the most common were mobile apps (86/126, 68.3%), desktop apps (73/126, 57.9%), telephone calls (49/126, 38.9%), and websites (45/126, 35.7%).

**Conclusions:**

Technologies played a crucial role in mitigating the challenges faced during the COVID-19 pandemic. We did not find papers describing the implementation of other technologies (eg, contact-tracing apps, drones, blockchain) during the first wave. Furthermore, technologies in this review were used for other purposes (eg, drugs and vaccines discovery, social distancing, and immunity passport). Future research on studies on these technologies and purposes is recommended, and further reviews are required to investigate technologies implemented in subsequent waves of the pandemic.

## Introduction

### Background

Since late 2019, COVID-19 has spread rapidly across the globe, leading to 175 million infections and nearly 3.8 million deaths worldwide [1]. Health care systems quickly became overwhelmed with the influx of patients, which led to overcrowded or inaccessible hospitals, delayed interventions for time-sensitive conditions and chronic illnesses, and overworked treatment teams that lacked proper personal protective equipment [2].

While developing strategic implementations to eradicate the disease, treatment providers, government entities, and individuals relied on technologies to mitigate some of the disastrous effects of COVID-19. Specifically, technologies have been used by treatment providers working with patients via telehealth; for contact tracing, screening, diagnosing COVID-19, establishing vaccination or immunity passports, and delivering foods, medications, and equipment; for predicting the trend of the pandemic, drug and vaccine discovery, social distancing; and for monitoring quarantined individuals [3]. Technological support played an integral role in guiding these forms of treatment and newly implemented policies likely prevented an even greater global crisis.

Despite the technological advancements introduced as a result of COVID-19, which some researchers have referred to as a “digital revolution,” more technologies are still required to alleviate the impact of COVID-19. For instance, hospital-at-home care has yet to be widely introduced, despite many patients who may benefit from this treatment [4]. While less-urgent telehealth visits have played a large role in keeping health care facilities at an appropriate patient capacity, the hospital-at-home model would allow for patients to receive treatment comfortably at home. Patients treated at home would likely not contribute to overcrowding or take up valuable personal protective equipment resources that treatment providers may need for more serious uses. Other researchers have commented that the use of artificial intelligence—which has grown instrumentally throughout the COVID-19 pandemic—has been sparing in assisting surgeons [5]. There is hope that, in the future, artificial intelligence can facilitate patient recovery by something as advanced as implementation in surgery or as simple as patient ventilatory parameter adjustment.

Overall, technologies have played an integral part in keeping communities safe and informed, giving treatment providers innovative ways to effectively perform their duties, and providing government administrations real-time information on the virus’ effects.

### Research Problem and Aim

Since the first COVID-19 infection was recorded in December 2019 [6], researchers have written extensively on how technology has played a crucial role in the fight against COVID-19—several reviews have been conducted about technologies used during the pandemic. For instance, Davalbhakta et al [7] reviewed mobile apps and assessed their quality, with the primary purpose of mitigating issues related to COVID-19. Gao et al [8] conducted a rapid review about the use of telemedicine for COVID-19 for any purpose. Another review [9] explored the COVID-19–related uses of the Internet of Things, unmanned aerial vehicles, blockchain, artificial intelligence, and 5G.

While these reviews [7-9] play a substantial role in informing how technologies have been used to control the spread of COVID-19, they have been limited by either their topic or their nature. Specifically, most reviews [3,7,8,10] only focused on one or a few technologies at a time, which is an approach that does not allow for gauging the all-encompassing scope of technology use during this pandemic. Moreover, some reviews were literature reviews; therefore, they were not systematic or comprehensive (ie, as systematic reviews and scoping reviews are) [11]. Lastly, some reviews included proposed technologies, rather than technologies that had been implemented [3,10]. In contrast, we aimed to provide an overview of technologies, as reported in the literature, that were implemented during the first wave of the COVID-19 pandemic.

## Methods

### Overview

To achieve the above-mentioned objective, while ensuring both replicable and transparent methods, we conducted a scoping review using PRISMA ((Preferred Reporting Items for Systematic Reviews and Meta-analyses) Extension for Scoping [12].

### Search Strategy

#### Search Sources

We searched the following electronic databases on August 14, 2020: MEDLINE (via Ovid), EMBASE (via Ovid), PsycInfo (via Ovid), Scopus, Web of Science, IEEE Xplore, ACM Library, and Google Scholar. To account for Google Scholar’s vast number of results, only the first 100 citations (sorted by relevance) were screened. To identify additional studies, eligible studies’ reference lists were checked, and papers that cited eligible studies were checked.

#### Search Terms

To develop the search queries (Multimedia Appendix 1), 2 experts in digital health were consulted and other systematic reviews of relevance to the review were checked. Terms were chosen based on the target intervention (ie, technologies) and the target disease (ie, COVID-19).

### Study Eligibility Criteria

We included papers that focused on technologies or digital tools implemented during COVID-19 to provide health-related services (eg, consultations, diagnosis, and follow-up) regardless of the target health condition, user, or setting. Studies that only proposed technologies (ie, that did not implement technologies) or focused on technologies for providing non–health-related services (eg, shopping, food delivery) were excluded. Peer-reviewed journal articles were considered, along with conference proceedings, theses, dissertations, preprints, and reports. We restricted our search to studies published between January 1, 2020 to August 14, 2020. Conference abstracts, proposals, editorials, commentaries, and non–English-language papers were excluded. No restrictions related to country of publication, outcome measure, and study design were applied.

### Study Selection

We followed 3 steps to identify the relevant papers: (1) duplicates were identified and removed using EndNote (Clarivate Analytics). (2) Reviewers (AH and IA) independently checked the titles and abstracts of all identified studies. (3) The 2 reviewers independently read the full texts of papers included from the second step. Any disagreements between the 2 reviewers were resolved by consulting a third reviewer (AA).

### Data Extraction and Synthesis

We designed a form to extract the data precisely and systematically (Multimedia Appendix 2). The form was pilot-tested using 10 eligible papers. Given the large number of eligible papers, the list was divided equally into 2 lists. While AA and MA independently extracted the data from the first list, AAA and US independently extracted data from the second list. Any disagreements between the reviewers were resolved by discussion until consensus was reached. A narrative approach was then used to synthesize the extracted data; tables were also used to describe technology types, aims, target users, target health conditions, settings, and modes. Excel (Microsoft Inc) was utilized to manage data extraction and synthesis.

## Results

### Search Results

**Figure 1:**
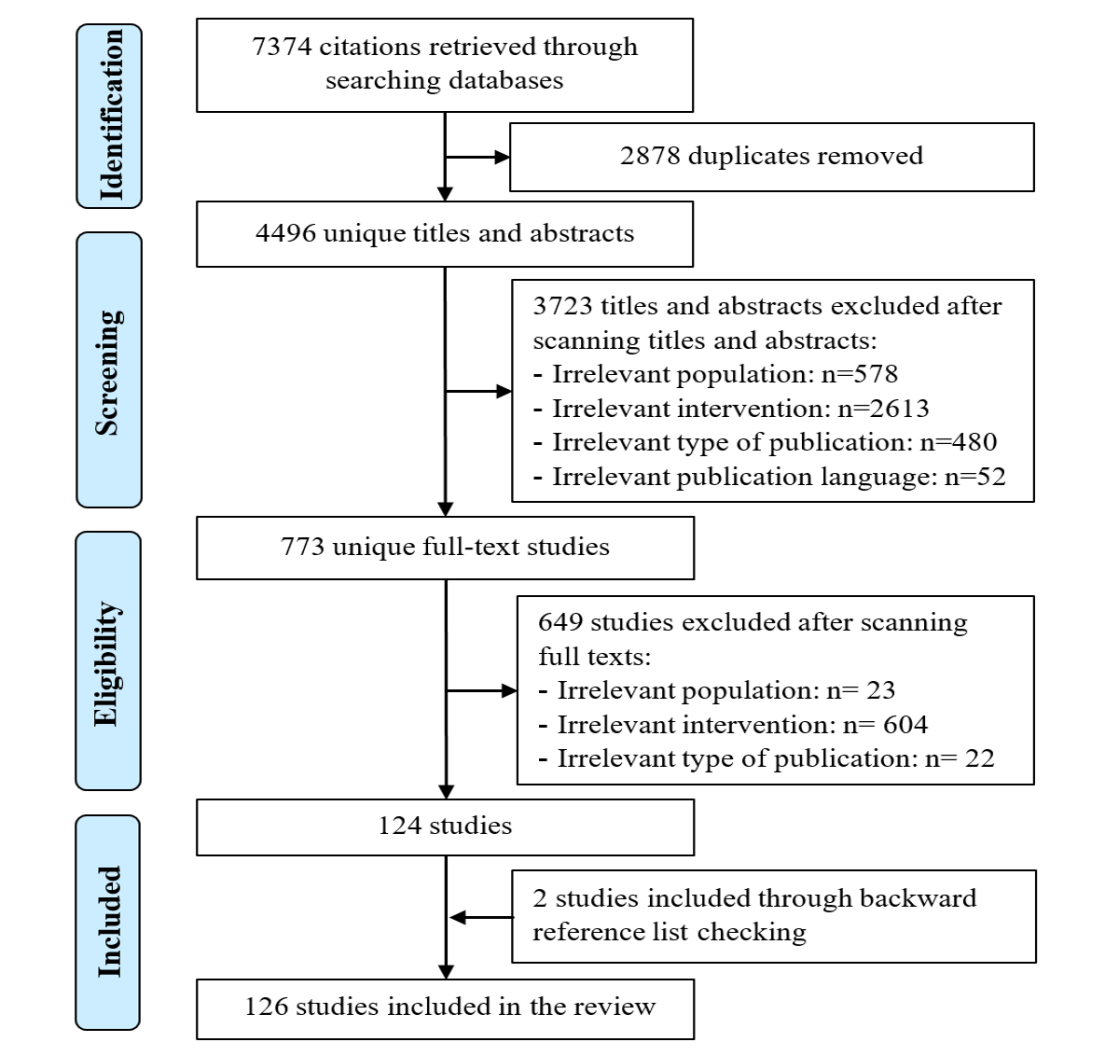
Flowchart of the study selection process.

Of 7374 citations retrieved through the search (Figure 1), 2878 duplicates were identified and removed. The remaining 4496 citation titles and abstracts were screened, of which 3723 citations were excluded. In total, 126 publications were included in this review [13-138].

### Characteristics of the Included Studies

The first paper [98] was published approximately 2 months after of the emergence of COVID-19. While only 1 paper [98] was published in the first 3 months, there was a sharp increase in the number published in the 5 subsequent months (Table 1); the largest number of studies (n=39) was published in June. The majority of studies (123/126, 97.6%) were papers in peer-reviewed journals, while the remaining studies were reports (n=2) and preprints (n=1). Studies were conducted in 23 different countries—the largest number of studies was carried out in the United States (51/126, 40.5%), followed by China (15/126, 11.9%), Italy (11/126, 8.7%), and India (10/126, 7.9%). Multimedia Appendix 3 shows the characteristics of each study.

**Table 1:** Characteristics of studies.

Characteristic		Studies (n=126), n (%)	Reference
**Month of publication**			
	February	1 (0.8)	[98]
	April	14 (11.1)	[26,41,52,54,55,62,69,77,91,102,105,114,117,122]
	May	31 (24.6)	[17,18,21,30,38,42,43,51,53,61,63-65,68,70,73,75,76,81,82,86,87,90,95,100,101,116,125,129,130,138]
	June	39 (31.0)	[13,20,22,24,25,27-29,31,32,34,36,37,40,44,46,48,50,56,58,67,72,78-80,83,84,89,94,96, 99,103,104,108,113,120,124,132,137]
	July	30 (23.8)	[15,16,23,33,35,39,45,47,49,57,59,66,71,74,85,88,93,97,106,107,109,112,123,126,128,131,133-136]
	August	11 (8.7)	[14,19,60,92,110,111,115,118,119,121,127]
**Type of publication**			
	Journal article	123 (97.6)	[13-74,76-110,112-120,122-138]
	Report	2 (1.6)	[75,111]
	Preprint	1 (0.8)	[121]
**Country of publication**			
	United States	51 (40.5)	[16,18,20,25,30,31,36,37,39,41,46,47,49,50,56,58,67,69,70,72-76,79,84,85,87,88,92,93, 97,102,105,108-115,117,121,125,126,129,133,134,136,137]
	China	15 (11.9)	[14,15,44,51,52,71,77,81,96,98,107,122,127,128,138]
	Italy	11 (8.7)	[19,27,34,45,55,60,62,65,90,99,100]
	India	10 (7.9)	[23,24,28,38,53,63,64,101,103,124]
	United Kingdom	7 (5.6)	[43,66,82,104,119,120,130]
	Canada	4 (3.2)	[42,78,91,95]
	Spain	4 (3.2)	[29,33,48,106]
	Germany	3 (2.4)	[32,123,132]
	Japan	3 (2.4)	[59,80,131]
	Australia	2 (1.6)	[35,61]
	Denmark	2 (1.6)	[21,40]
	Netherlands	2 (1.6)	[86,94]
	Poland	2 (1.6)	[22,68]
	Argentina	1 (0.8)	[54]
	Austria	1 (0.8)	[17]
	Bahrain	1 (0.8)	[13]
	Brazil	1 (0.8)	[116]
	Egypt	1 (0.8)	[57]
	Mexico	1 (0.8)	[26]
	Serbia	1 (0.8)	[83]
	Singapore	1 (0.8)	[118]
	South Africa	1 (0.8)	[135]
	Taiwan	1 (0.8)	[89]

### Characteristics of Technologies

The 126 studies featured 11 different types of technologies used during the first wave of COVID-19 (Table 2). The most common type was telemedicine, which was used in approximately 84.9% of the studies (107/126). The mode of telemedicine was synchronous in 83 studies, asynchronous in 8 studies, and both in 17 studies. Technologies (Multimedia Appendix 4) were used for 20 different purposes. The most common aims of the technologies were providing consultations (75/126, 59.5%), following up with patients (45/126, 35.7%), and monitoring patient health status (22/126, 17.4%).

**Table 2:** Characteristics of technologies in the studies.

Characteristics			Studies (n=126), n (%)		Reference
**Technology type^a^**					
	Telemedicine	107 (84.9)		[13-119]	
	Clinical decision support tools	11 (8.7)		[78,80-83,88,89,117,120-122]	
	Robotic systems	6 (4.8)		[123-128]	
	Symptom trackers	6 (4.8)		[78,122,129-132]	
	Dashboards	5 (4.0)		[78,93,117,133,134]	
	Electronic health records	4 (3.2)		[44,117,132,135]	
	Patient portals	3 (2.4)		[76,83,117]	
	Educational platforms	2 (1.6)		[136,137]	
	Triage tools	1 (0.8)		[93]	
	Reporting system	1 (0.8)		[83]	
	Low-dose computed tomography	1 (0.8)		[138]	
**Mode of telemedicine^b^**					
	Synchronous	83 (76.9)		[13-22,25,26,28,30,31,33,34,36-45,47,49-51,54-59,62-64,66-76,78,79,82-89,92,93,96,97, 99-103,105,107-116,118,119]	
	Asynchronous	8 (7.4)		[23,24,48,81,91,94,98,129]	
	Both	17 (15.7)		[27,29,32,35,46,52,53,60,61,65,77,80,90,95,104,106,117]	
**Technology aim^a^**					
	Consultation	75 (59.5)		[13-87]	
	Follow up	45 (35.7)		[50-81,90,95-102,106,107,116,138]	
	Monitoring health status	22 (17.4)		[78,85-87,93,94,103-108,117,122,126-132,134]	
	Education	14 (11.1)		[46,47,49-52,77,91-94,107,136,137]	
	Triage	12 (9.5)		[52,78,79,82-84,87-90,93,117]	
	Treatment	12 (9.5)		[47,79,85,86,110-116,125]	
	Diagnosing	8 (6.3)		[45-47,89,117,126-128]	
	Screening	4 (3.2)		[80,81,109,124]	
	Accessing patient records	4 (3.2)		[44,117,132,135]	
	Monitoring health services	4 (3.2)		[93,117,133,134]	
	Decision making	3 (2.4)		[117,120,122]	
	Generating reports	3 (2.4)		[78,83,117]	
	Clinical assessment	2 (1.6)		[92,119]	
	Administrative support	2 (1.6)		[118,119]	
	Medical data exchange	2 (1.6)		[83,117]	
	Connecting patients and families	2 (1.6)		[93,108]	
	Booking appointments	2 (1.6)		[78,117]	
	Drug delivery	1 (0.8)		[48]	
	Prognosis	1 (0.8)		[121]	
	Personal assistant	1 (0.8)		[123]	
**Technology development^a,c^**					
	Built for purpose	46 (58.2)		[20,25,29,34,41,44,45,47,49,51,56,58,61,69,70,73,76,80,81,83,86,89-91,93-95,101,104-107,114, 115,119,120,122,123,125-128,131,132,135,136]	
	Purpose-shifted	45 (57.0)		[14-16,18,20,23-26,31,34,37-39,47,50,53,54,57,62-64,67,69-72,74-77,87,90,96,97,100,101,107, 108,111,113,115,117,120,137]	
**Social media and videoconferencing platforms^a^**					
	Zoom	22 (17.5)		[16,25,31,34,37,50,57,62,67,69,70,72,74-77,87,108,111,113,115,137]	
	WhatsApp	12 (9.5)		[23-25,38,53,54,57,62,63,90,100,120]	
	FaceTime	6 (4.8)		[20,72,76,97,101,108]	
	WeChat	5 (4.0)		[14,15,71,96,107]	
	WebEx	5 (4.0)		[47,64,108,111,115]	
	Google Duo	5 (4.0)		[20,25,62,76,101]	
	Skype	4 (3.2)		[26,62,76,100]	
	Telegram	3 (2.4)		[25,62,90]	
	Messenger	2 (1.6)		[25,90]	
**Target users**					
	Health consumers and health care professionals	103 (81.7)		[13-32,34-41,43,45-60,62-84,86-93,95-102,104-118,123,131,132,138]	
	Health care professionals	15 (11.9)		[33,42,44,59,85,103,119,121,125-128,135-137]	
	Health consumers	5 (4.0)		[61,94,124,129,130]	
	Decision makers	3 (2.4)		[122,133,134]	
**Target conditions^a^**					
	COVID-19	38 (30.2)		[26,29,44,45,52,75,78,81,85,88,89,93-95,103,104,106-109,117-119,121,122,124,126-136,138]	
	Any conditions	21 (16.7)		[15,18,25,28,35,40,48,49,54,55,73,79,80,83,84,90,100,102,105,123,137]	
	Mental conditions	13 (10.3)		[31,33,42,50,74,76,87,91,111-115]	
	Cardiovascular conditions	9 (7.1)		[14,61,65,68,86,96,97,120,125]	
	Cancer conditions	6 (4.8)		[16,32,46,66,77,82]	
	Neurological conditions	6 (4.8)		[20,23,30,58,62,101]	
	Prenatal and postnatal conditions	6 (4.8)		[41,47,56,59,60,110]	
	Diabetes	5 (4.0)		[13,17,21,37,99]	
	Orthodontic conditions	4 (3.2)		[39,43,69,70]	
	Rheumatic conditions	4 (3.2)		[22,27,38,63]	
	Ophthalmic conditions	3 (2.4)		[24,53,71]	
	Urologic conditions	3 (2.4)		[72,92,134]	
	Dermatological conditions	3 (2.4)		[19,57,64]	
	Ear, nose, and throat conditions	2 (1.6)		[67,116]	
	Liver conditions	2 (1.6)		[36,98]	
	Gastrointestinal conditions	1 (0.8)		[36,91]	
	Orthodontic conditions	1 (0.8)		[34]	
	Reproductive conditions	1 (0.8)		[87]	
	Transplant conditions	1 (0.8)		[51]	
**Setting^a^**					
	Hospitals	81 (64.3)		[13-15,17,18,21-23,27-35,37,41-44,48,50-60,64,66,69-73,77,79,80,82,85,88-90,92-94,96-100, 102-108,110,116-123,126-128,133-135,137,138]	
	Medical clinics	46 (36.5)		[16,19,20,24-26,36,38-41,45-47,49,52,57,61-63,65,67,68,74-76,83,84,86-88,93,95,101,109,111-115, 117,123,125,131,132]	
	Community	7 (5.6)		[78,91,123,124,129,130,136]	
**Internet connectivity^a^**					
	Web-based	106 (84.1)		[13-16,18-20,23-32,34-39,41,43-54,56-58,60-65,67,69-81,83-90,92-98,100-115,117,119,120,122, 125-133,135-137]	
	Non–web-based	63 (50)		[13,17,20-24,26-33,35,40,42,43,46,49,52,53,55,59-62,65-68,74,76,77,79,81,82,84,88-93,95,97,99,101, 102,104,106,112,115-118,120,121,123,124,134,138]	
**Modes^a^**					
	Mobile apps	86 (68.3)		[13-16,18-20,23-26,28,30-32,34,36-39,41,43-45,47,49,50,53,54,56,57,61-65,67,69-78,80,81,84-88,90, 92-94,96-98,100-115,117,120,122,129-132,135,137]	
	Desktop apps	73 (57.9)		[14,16,19,20,23-26,28-32,34-38,47,50,52-54,57,58,62-65,67,69-78,80,82,83,85,87-90,92,93,96,100, 101,105,107-111,113-115,119-121,131,133,134,137,138]	
	Telephone calls	49 (38.9)		[13,17,20-24,27-33,35,40,42,43,46,49,52,53,55,59-62,65-68,76,77,79,82,84,90,92,97,99,101, 102,104,106,112,115-118]	
	Websites	45 (35.7)		[14-16,20,23-26,31,34,37,38,46,47,50,51,53,57,62-64,67,69-72,74-77,83,87,90,95,96,100,101,107, 108,111,113,115,120,136,137]	
	Emails	13 (10.3)		[27,29,32,35,46,53,57,60,65,77,90,95,117]	
	Robot	6 (4.8)		[123-128]	
	Text messages	2 (1.6)		[48,91]	
	Interphone	1 (0.8)		[26]	
	Automated vital-sign monitor	1 (0.8)		[93]	
	Closed-circuit television cameras	1 (0.8)		[103]	
	Headset	1 (0.8)		[119]	

^a^Numbers do not add to total.

^b^Number of telemedicine studies (n=108) was used to calculate percentages.

^c^We were able to identify the type of technology development in 79 studies; therefore, we used this number to calculate percentages.

The type of technology development was identified in 79 studies (Table 2). Technologies in these 79 studies were designed to provide the respective purpose from the beginning (46/79, 58.2%), or used for other purposes rather than the purpose for which they had been developed (45/79, 57.0%). Studies utilized 8 social media and videoconferencing platforms. The most common platforms were Zoom (22/126, 17.5%) and WhatsApp (12/126, 9.5%). The target users in the studies were health care professionals (15/126, 11.9%), health consumers (5/126, 4%), health care professionals as well as health consumers (103/126, 81.7%), and decision makers (3/126, 2.4%). Technologies in the studies targeted 19 groups of health conditions. The most targeted health condition by the technologies was COVID-19 (38/126, 30.2%), followed by any physical health conditions (21/126, 16.7%) and mental health conditions (13/126, 10.3%). Multimedia Appendix 5 shows characteristics of the technologies used for COVID-19.

Technologies provided services to individuals in hospitals in 64.3% (81/126), medical clinics in 36.5% (46/126), and the community in 5.6% (7/126) of the studies. Technologies were web-based in 84.1% of the studies (106/126) and non–web-based in half of the included studies. Technologies were used through 11 modes. The most common mode was mobile apps (86/126, 68.3%), followed by desktop apps (73/126, 57.9%), telephone calls (49/126, 38.9%), and websites (45/126, 35.7%).

## Discussion

### Principal Findings

This review features all technologies utilized in the first efforts to provide health care services for all kinds of clinical conditions during the pandemic, which ideally provides a holistic perspective of technology use during the initial stages of the COVID-19 pandemic. Only 1 paper [98] was published in the first 3 months of the pandemic, whereas the number of the studies significantly increased in subsequent months. This may be attributed to the fact that the process of developing technologies, writing a report on its use, and publishing the report likely require at least 3 months. Furthermore, experts suggest that evidence-based technology adoption is critical for designing, using, and implementing digital tools in the fight against COVID-19 [139]. Most technologies were implemented in the United States. This is expected given that the United States is both the country most infected by COVID-19 [1] as well as highly advanced in technologies.

As evident from the results, telemedicine was the most frequently utilized technology in papers published from January to August 2020. One of the main challenges during the first few months of the pandemic was providing necessary health care services while simultaneously mitigating the risk of infection for patients and health care workers from inadequate supplies of personal protective equipment. The majority of studies included in this review reported the use of synchronous telemedicine, which can improve access to specialized care services at familiar settings (ie, homes) in a more time- and cost-effective manner [140,141]. Similar to earlier reviews, our results showed that telemedicine was used for COVID-19 screening, providing health care advice, and mental health therapy [8]. The remainder of the technologies reported in the studies involved the use of clinical decision support tools, robotic systems, and symptom trackers. While many other technologies were implemented during the first wave of COVID-19 (eg, contact-tracing apps and drones [11]), our review did not report any. This could be attributed to the fact that (1) there are no publications on each technology implemented during COVID-19, (2) only studies published in English were included in this review, and (3) only academic literature was searched.

Several social media and videoconferencing platforms were used in the first wave. Zoom was the most commonly used; this may be attributed to the fact that Zoom is one of the Health Insurance Portability and Accountability Act–compliant teleconferencing platforms and has been integrated into electronic health record systems in many health care settings [31,67,87]. This review also found that most of the implemented technologies targeted both health care providers and health consumers. This is likely because extreme measures (eg, lockdowns; curfews; and closures of many businesses, spaces, and organizations) imposed to prevent the spread of COVID-19 immensely disrupted the routine delivery of health care services to many patients—especially those with chronic conditions—therefore, technologies were required to keep patients and health care providers connected.

COVID-19 was the most targeted health condition by the technologies. This can be attributed to the fact that COVID-19 was given priority over other health conditions in the first wave, when there were extremely large numbers of daily infections, and no treatment or vaccine was available. Mobile apps were the most common venue used to target illness during this time period, as well. This is reasonable given the widespread use of mobile devices over the world [142].

### Research and Practical Implications

Modern medicine relies on evidence-based practices and interventions. Many researchers argue that digital health tools and technologies are not exempt and should be evaluated and validated to demonstrate their efficacy [143]. Widespread technology adoption relies heavily upon the availability of evidence regarding technology’s effectiveness [144]. While there is a sense of urgency because of the COVID-19 pandemic and its impact on every aspect of daily living, evaluation of digital tools’ and technologies’ effectiveness remains paramount. However, since the COVID-19 pandemic is still ongoing, there are copious amounts of digital tools and technologies that need to be peer-reviewed, undergo rigorous testing, and be integrated into public health systems [11].

Many digital interventions have failed to be integrated into national systems due to low levels of acceptance at that scale; many digital interventions have been successful at pilot scales only [145,146]. Therefore, it is integral to carefully evaluate digital health tools and technologies by providing validated, documented, and reproducible clinically meaningful evidence [143,147]. There is a need for more peer-reviewed evidence-based research into the effectiveness of these digital tools and technologies.

Overall, there is emerging consensus that digital tools and technologies play a central role in public health interventions during public health outbreaks (eg, the COVID-19 pandemic) by complementing and augmenting traditional or nondigital interventions [11]. However, despite the promise of technologies’ benefits, such as alleviating the impact of COVID-19 (such as providing access to health care services during lockdowns through telemedicine), solely relying on these technologies may widen disparities and exacerbate existing vulnerabilities of those who are unable to adopt or afford these technologies [148]. These digital tools and technologies pose the risk of generating informatics-based (or digital) inequalities that disproportionately favor groups with socioeconomic advantages [149]. Therefore, health system leaders, informaticians, and policy makers need to consider and act upon underlying factors contributing to inequalities in terms of access to resources, digital literacy, and usability [150].

To ensure sustainable investments, use, and adoption in digital tools and technologies after the COVID-19 pandemic has been resolved, issues related to regulating and reimbursing the use of these tools and technologies need to be addressed [151]. Additionally, it is paramount that it is not assumed that digital tools and technologies will naturally become integrated into routine clinical practice post–COVID-19; participatory approaches that include all stakeholders are essential to sustaining the use and adoption of digital tools and technologies [152].

### Strengths and Limitations

#### Strengths

To the best of our knowledge, this paper is the first review to explore all technologies implemented to provide health care services during COVID-19. This review can be considered comprehensive as it does not focus on specific technologies, diseases, users, settings, or countries. Therefore, the review provides a holistic view of the role of technologies during COVID-19 to help decision makers, health care providers, and health care consumers understand the potentials of technologies.

Given that we followed well-recommended guidelines in developing, executing, and reporting this review, it can be considered a robust and high-quality review. The search was sensitive and precise because the most popular databases in health and information technology were searched using a well-developed search query.

The risk of publication bias is minimal in this review because of the strategies that were used, such as searching grey literature databases (ie, Google Scholar) and backward and forward reference list checking. Furthermore, this review has a low risk of selection bias because study selection and data extraction were conducted by 2 reviewers independently.

#### Limitations

Although the search query consisted of 89 terms related to technologies, it is possible that other terms related to technologies were missed. Therefore, it is likely that several relevant studies were not included. Because of practical constraints, the search was restricted to English studies. Therefore, several studies written in other languages were not included.

This review focused on implemented technologies and excluded proposed technologies, which perhaps could have been subsequently implemented. Thus, several important technologies were not considered in our review. Although this review shows the potential of technologies, it cannot comment on their effectiveness, as it is beyond the scope.

### Conclusion

Technologies played a crucial role in mitigating challenges arising from the COVID-19 pandemic, and in the first wave, numerous technologies were used for various purposes. However, we did not find papers on other technologies such as contact-tracing apps, drones, blockchain, unmanned aerial vehicles, wearable devices, and personal protective equipment that were implemented during the first wave of the pandemic. Furthermore, technologies reported in the studies were used for other purposes, such as drug and vaccine discovery, social distancing, and immunity passports. Therefore, it is recommended that researchers conduct studies on these technologies and purposes. Further reviews are required about technologies implemented in subsequent waves of the COVID-19 pandemic. There is also a need for more peer-reviewed evidence-based research into the effectiveness of these digital tools and technologies and users’ satisfaction.

## Appendix

Multimedia Appendix 1 Search strategy.

Multimedia Appendix 2 Data extraction form.

Multimedia Appendix 3 Characteristics of each included study.

Multimedia Appendix 4 Characteristics of technologies in each included study.

Multimedia Appendix 5 Characteristics of technologies used for COVID-19, as reported in 38 studies.

## Abbreviations

PRISMA: Preferred Reporting Items for Systematic Reviews and Meta-analyses
