# Supplementary material for: Overview of Technologies Implemented During the First Wave of the COVID-19 Pandemic: Scoping Review
Source: J Med Internet Res. 2021 Sep 14;23(9):e29136. doi: 10.2196/29136 (PMC8767979; doi:10.2196/29136)
Supplement: Multimedia Appendix 1 [file jmir_v23i9e29136_app1.docx]

**Appendix 1: Search strategy**

Database(s): **Ovid MEDLINE(R) and Epub Ahead of Print, In-Process & Other Non-Indexed Citations and Daily**1946 to August 14, 2020
Search Strategy:

| **#** | **Searches** | **Results** |
| --- | --- | --- |
| 1 | Health information technolog*.tw. | 2951 |
| 2 | e-health.tw. | 2125 |
| 3 | ehealth.tw. | 2639 |
| 4 | "digital health".tw. | 1455 |
| 5 | exp Telemedicine/ | 29202 |
| 6 | telemedicine.tw. | 10768 |
| 7 | tele-medicine.tw. | 109 |
| 8 | telehealth.tw. | 4312 |
| 9 | tele-health.tw. | 110 |
| 10 | telecare.tw. | 648 |
| 11 | tele-care.tw. | 31 |
| 12 | telepsychiatr*.tw. | 595 |
| 13 | "tele-psychiatr*".tw. | 16 |
| 14 | teledermatology.tw. | 769 |
| 15 | tele-dermatology.tw. | 13 |
| 16 | teleconsultation.tw. | 858 |
| 17 | "tele-consultation".tw. | 54 |
| 18 | teleconferenc*.tw. | 1153 |
| 19 | "tele-conferenc*".tw. | 19 |
| 20 | telesurger*.tw. | 242 |
| 21 | "tele-surger*".tw. | 29 |
| 22 | exp Electronic Prescribing/ | 1029 |
| 23 | "electronic prescribing".tw. | 672 |
| 24 | "e-prescribing".tw. | 404 |
| 25 | exp Medical Order Entry Systems/ | 2258 |
| 26 | "computerized provider order entry".tw. | 426 |
| 27 | "computerised provider order entry".tw. | 38 |
| 28 | CPOE.tw. | 1064 |
| 29 | exp Patient Portals/ | 378 |
| 30 | "patient portal*".tw. | 936 |
| 31 | exp Health Records, Personal/ | 1945 |
| 32 | "personal health record*".tw. | 992 |
| 33 | exp Electronic Health Records/ | 20386 |
| 34 | "electronic health record*".tw. | 15415 |
| 35 | "electronic medical record*".tw. | 16470 |
| 36 | kiosk*.tw. | 406 |
| 37 | "mobile health".tw. | 3580 |
| 38 | mhealth.tw. | 3087 |
| 39 | m-health.tw. | 383 |
| 40 | exp Mobile Applications/ | 6023 |
| 41 | "mobile app*".tw. | 4172 |
| 42 | "smartphone app*".tw. | 3155 |
| 43 | "smart phone app*".tw. | 179 |
| 44 | exp Decision Support Systems, Clinical/ | 7998 |
| 45 | "decision support system*".tw. | 5476 |
| 46 | exp Artificial Intelligence/ | 98383 |
| 47 | exp Machine Learning/ | 19282 |
| 48 | "artificial intelligence".tw. | 7528 |
| 49 | "machine learning".tw. | 28471 |
| 50 | exp Deep Learning/ | 2562 |
| 51 | "deep learning".tw. | 9399 |
| 52 | "neural network".tw. | 32015 |
| 53 | exp Wearable Electronic Devices/ | 11769 |
| 54 | wearable*.tw. | 12245 |
| 55 | sensor*.tw. | 359705 |
| 56 | "digital tool*".tw. | 459 |
| 57 | "digital device*".tw. | 401 |
| 58 | exp Robotics/ | 28102 |
| 59 | robot*.tw. | 44787 |
| 60 | dashboard*.tw. | 1258 |
| 61 | digital thermometer*.tw. | 235 |
| 62 | "thermal camera*".tw. | 344 |
| 63 | exp Virtual Reality/ | 1948 |
| 64 | "virtual reality".tw. | 9632 |
| 65 | exp Augmented Reality/ | 122 |
| 66 | "augmented reality".tw. | 1979 |
| 67 | "contact tracing tool*".tw. | 0 |
| 68 | "contact tracing app*".tw. | 30 |
| 69 | "contact tracing device*".tw. | 0 |
| 70 | chatbot*.tw. | 114 |
| 71 | "chat-bot*".tw. | 17 |
| 72 | "chat bot*".tw. | 17 |
| 73 | "dialogue agent*".tw. | 4 |
| 74 | "conversational agent*".tw. | 117 |
| 75 | drone*.tw. | 1689 |
| 76 | "computer vision".tw. | 3601 |
| 77 | exp Text Messaging/ | 2918 |
| 78 | "instant messag*".tw. | 324 |
| 79 | "text messag*".tw. | 4155 |
| 80 | exp Social Media/ | 8027 |
| 81 | "social media".tw. | 11185 |
| 82 | Twitter.tw. | 3127 |
| 83 | Facebook.tw. | 3592 |
| 84 | Instagram.tw. | 535 |
| 85 | Youtube.tw. | 1919 |
| 86 | Whatsapp.tw. | 471 |
| 87 | Wechat.tw. | 290 |
| 88 | "Tik ToK".tw. | 2 |
| 89 | Snapchat.tw. | 86 |
| 90 | "novel coronavirus".tw. | 3824 |
| 91 | "coronavirus 2019".tw. | 701 |
| 92 | "novel corona virus".tw. | 121 |
| 93 | "corona virus 2019".tw. | 20 |
| 94 | "COVID 2019".tw. | 108 |
| 95 | "COVID 19".tw. | 36141 |
| 96 | "COVID19".tw. | 366 |
| 97 | "COVID-19".tw. | 36141 |
| 98 | "SARS-CoV-2".tw. | 10722 |
| 99 | "HCoV-19".tw. | 12 |
| 100 | "2019-nCoV".tw. | 802 |
| 101 | "severe acute respiratory syndrome coronavirus 2".tw. | 3605 |
| 102 | "Wuhan coronavirus".tw. | 18 |
| 103 | "Wuhan corona virus".tw. | 0 |
| 104 | "Chinese coronavirus".tw. | 2 |
| 105 | 1 or 2 or 3 or 4 or 5 or 6 or 7 or 8 or 9 or 10 or 11 or 12 or 13 or 14 or 15 or 16 or 17 or 18 or 19 or 20 or 21 or 22 or 23 or 24 or 25 or 26 or 27 or 28 or 29 or 30 or 31 or 32 or 33 or 34 or 35 or 36 or 37 or 38 or 39 or 40 or 41 or 42 or 43 or 44 or 45 or 46 or 47 or 48 or 49 or 50 or 51 or 52 or 53 or 54 or 55 or 56 or 57 or 58 or 59 or 60 or 61 or 62 or 63 or 64 or 65 or 66 or 67 or 68 or 69 or 70 or 71 or 72 or 73 or 74 or 75 or 76 or 77 or 78 or 79 or 80 or 81 or 82 or 83 or 84 or 85 or 86 or 87 or 88 or 89 | 661121 |
| 106 | 90 or 91 or 92 or 93 or 94 or 95 or 96 or 97 or 98 or 99 or 100 or 101 or 102 or 103 or 104 | 40610 |
| 107 | 105 and 106 | 2647 |
| 108 | limit 107 to yr="2020 -Current" | 2645 |
| 109 | limit 108 to english language | 2586 |

Database(s): **Embase**1974 to 2020 August 14
Search Strategy:

| **#** | **Searches** | **Results** |
| --- | --- | --- |
| 1 | Health information technolog*.tw. | 3112 |
| 2 | e-health.tw. | 2949 |
| 3 | ehealth.tw. | 2955 |
| 4 | "digital health".tw. | 1753 |
| 5 | exp Telemedicine/ | 41253 |
| 6 | telemedicine.tw. | 14606 |
| 7 | tele-medicine.tw. | 228 |
| 8 | telehealth.tw. | 5396 |
| 9 | tele-health.tw. | 256 |
| 10 | telecare.tw. | 773 |
| 11 | tele-care.tw. | 47 |
| 12 | telepsychiatr*.tw. | 727 |
| 13 | "tele-psychiatr*".tw. | 38 |
| 14 | teledermatology.tw. | 1137 |
| 15 | tele-dermatology.tw. | 33 |
| 16 | teleconsultation.tw. | 1082 |
| 17 | "tele-consultation".tw. | 115 |
| 18 | teleconferenc*.tw. | 1830 |
| 19 | "tele-conferenc*".tw. | 42 |
| 20 | telesurger*.tw. | 287 |
| 21 | "tele-surger*".tw. | 50 |
| 22 | exp Electronic Prescribing/ | 3037 |
| 23 | "electronic prescribing".tw. | 1374 |
| 24 | "e-prescribing".tw. | 593 |
| 25 | exp Medical Order Entry Systems/ | 252 |
| 26 | "computerized provider order entry".tw. | 511 |
| 27 | "computerised provider order entry".tw. | 40 |
| 28 | CPOE.tw. | 1589 |
| 29 | exp Patient Portals/ | 248625 |
| 30 | "patient portal*".tw. | 1287 |
| 31 | exp Health Records, Personal/ | 248625 |
| 32 | "personal health record*".tw. | 1159 |
| 33 | exp Electronic Health Records/ | 18523 |
| 34 | "electronic health record*".tw. | 22408 |
| 35 | "electronic medical record*".tw. | 35943 |
| 36 | kiosk*.tw. | 567 |
| 37 | "mobile health".tw. | 3743 |
| 38 | mhealth.tw. | 3073 |
| 39 | m-health.tw. | 540 |
| 40 | exp Mobile Applications/ | 12273 |
| 41 | "mobile app*".tw. | 5584 |
| 42 | "smartphone app*".tw. | 4413 |
| 43 | "smart phone app*".tw. | 455 |
| 44 | exp Decision Support Systems, Clinical/ | 3168 |
| 45 | "decision support system*".tw. | 6951 |
| 46 | exp Artificial Intelligence/ | 40673 |
| 47 | exp Machine Learning/ | 212948 |
| 48 | "artificial intelligence".tw. | 10004 |
| 49 | "machine learning".tw. | 35676 |
| 50 | exp Deep Learning/ | 8871 |
| 51 | "deep learning".tw. | 11815 |
| 52 | "neural network".tw. | 39433 |
| 53 | exp Wearable Electronic Devices/ | 3833 |
| 54 | wearable*.tw. | 13947 |
| 55 | sensor*.tw. | 422150 |
| 56 | "digital tool*".tw. | 691 |
| 57 | "digital device*".tw. | 534 |
| 58 | exp Robotics/ | 39327 |
| 59 | robot*.tw. | 70208 |
| 60 | dashboard*.tw. | 2401 |
| 61 | digital thermometer*.tw. | 323 |
| 62 | "thermal camera*".tw. | 463 |
| 63 | exp Virtual Reality/ | 17354 |
| 64 | "virtual reality".tw. | 12763 |
| 65 | exp Augmented Reality/ | 439 |
| 66 | "augmented reality".tw. | 2428 |
| 67 | "contact tracing tool*".tw. | 2 |
| 68 | "contact tracing app*".tw. | 30 |
| 69 | "contact tracing device*".tw. | 0 |
| 70 | chatbot*.tw. | 134 |
| 71 | "chat-bot*".tw. | 26 |
| 72 | "chat bot*".tw. | 26 |
| 73 | "dialogue agent*".tw. | 2 |
| 74 | "conversational agent*".tw. | 119 |
| 75 | drone*.tw. | 2069 |
| 76 | "computer vision".tw. | 3937 |
| 77 | exp Text Messaging/ | 5062 |
| 78 | "instant messag*".tw. | 418 |
| 79 | "text messag*".tw. | 5400 |
| 80 | exp Social Media/ | 21063 |
| 81 | "social media".tw. | 15745 |
| 82 | Twitter.tw. | 4439 |
| 83 | Facebook.tw. | 5641 |
| 84 | Instagram.tw. | 802 |
| 85 | Youtube.tw. | 2619 |
| 86 | Whatsapp.tw. | 764 |
| 87 | Wechat.tw. | 377 |
| 88 | "Tik ToK".tw. | 3 |
| 89 | Snapchat.tw. | 132 |
| 90 | "novel coronavirus".tw. | 3815 |
| 91 | "coronavirus 2019".tw. | 670 |
| 92 | "novel corona virus".tw. | 140 |
| 93 | "corona virus 2019".tw. | 23 |
| 94 | "COVID 2019".tw. | 116 |
| 95 | "COVID 19".tw. | 35926 |
| 96 | "COVID19".tw. | 374 |
| 97 | "COVID-19".tw. | 35926 |
| 98 | "SARS-CoV-2".tw. | 10161 |
| 99 | "HCoV-19".tw. | 12 |
| 100 | "2019-nCoV".tw. | 777 |
| 101 | "severe acute respiratory syndrome coronavirus 2".tw. | 3455 |
| 102 | "Wuhan coronavirus".tw. | 14 |
| 103 | "Wuhan corona virus".tw. | 0 |
| 104 | "Chinese coronavirus".tw. | 3 |
| 105 | 1 or 2 or 3 or 4 or 5 or 6 or 7 or 8 or 9 or 10 or 11 or 12 or 13 or 14 or 15 or 16 or 17 or 18 or 19 or 20 or 21 or 22 or 23 or 24 or 25 or 26 or 27 or 28 or 29 or 30 or 31 or 32 or 33 or 34 or 35 or 36 or 37 or 38 or 39 or 40 or 41 or 42 or 43 or 44 or 45 or 46 or 47 or 48 or 49 or 50 or 51 or 52 or 53 or 54 or 55 or 56 or 57 or 58 or 59 or 60 or 61 or 62 or 63 or 64 or 65 or 66 or 67 or 68 or 69 or 70 or 71 or 72 or 73 or 74 or 75 or 76 or 77 or 78 or 79 or 80 or 81 or 82 or 83 or 84 or 85 or 86 or 87 or 88 or 89 | 1111469 |
| 106 | 90 or 91 or 92 or 93 or 94 or 95 or 96 or 97 or 98 or 99 or 100 or 101 or 102 or 103 or 104 | 40323 |
| 107 | 105 and 106 | 3781 |
| 108 | limit 107 to yr="2020 -Current" | 3774 |
| 109 | limit 108 to english language | 3685 |
| 110 | limit 109 to exclude medline journals | 477 |

Database(s): **APA PsycInfo**1806 to August Week 2 2020
Search Strategy:

| **#** | **Searches** | **Results** |
| --- | --- | --- |
| 1 | Health information technolog*.tw. | 702 |
| 2 | e-health.tw. | 976 |
| 3 | ehealth.tw. | 1036 |
| 4 | "digital health".tw. | 310 |
| 5 | exp Telemedicine/ | 8816 |
| 6 | telemedicine.tw. | 2102 |
| 7 | tele-medicine.tw. | 17 |
| 8 | telehealth.tw. | 1733 |
| 9 | tele-health.tw. | 52 |
| 10 | telecare.tw. | 234 |
| 11 | tele-care.tw. | 11 |
| 12 | telepsychiatr*.tw. | 540 |
| 13 | "tele-psychiatr*".tw. | 13 |
| 14 | teledermatology.tw. | 45 |
| 15 | tele-dermatology.tw. | 0 |
| 16 | teleconsultation.tw. | 122 |
| 17 | "tele-consultation".tw. | 6 |
| 18 | teleconferenc*.tw. | 468 |
| 19 | "tele-conferenc*".tw. | 10 |
| 20 | telesurger*.tw. | 14 |
| 21 | "tele-surger*".tw. | 0 |
| 22 | exp Electronic Prescribing/ | 0 |
| 23 | "electronic prescribing".tw. | 56 |
| 24 | "e-prescribing".tw. | 30 |
| 25 | exp Medical Order Entry Systems/ | 0 |
| 26 | "computerized provider order entry".tw. | 24 |
| 27 | "computerised provider order entry".tw. | 2 |
| 28 | CPOE.tw. | 46 |
| 29 | exp Patient Portals/ | 0 |
| 30 | "patient portal*".tw. | 204 |
| 31 | exp Health Records, Personal/ | 0 |
| 32 | "personal health record*".tw. | 213 |
| 33 | exp Electronic Health Records/ | 832 |
| 34 | "electronic health record*".tw. | 1902 |
| 35 | "electronic medical record*".tw. | 1821 |
| 36 | kiosk*.tw. | 201 |
| 37 | "mobile health".tw. | 898 |
| 38 | mhealth.tw. | 991 |
| 39 | m-health.tw. | 137 |
| 40 | exp Mobile Applications/ | 894 |
| 41 | "mobile app*".tw. | 1559 |
| 42 | "smartphone app*".tw. | 884 |
| 43 | "smart phone app*".tw. | 70 |
| 44 | exp Decision Support Systems, Clinical/ | 0 |
| 45 | "decision support system*".tw. | 2010 |
| 46 | exp Artificial Intelligence/ | 21398 |
| 47 | exp Machine Learning/ | 9595 |
| 48 | "artificial intelligence".tw. | 5255 |
| 49 | "machine learning".tw. | 6670 |
| 50 | exp Deep Learning/ | 0 |
| 51 | "deep learning".tw. | 1346 |
| 52 | "neural network".tw. | 9966 |
| 53 | exp Wearable Electronic Devices/ | 0 |
| 54 | wearable*.tw. | 1299 |
| 55 | sensor*.tw. | 97710 |
| 56 | "digital tool*".tw. | 479 |
| 57 | "digital device*".tw. | 348 |
| 58 | exp Robotics/ | 7680 |
| 59 | robot*.tw. | 8292 |
| 60 | dashboard*.tw. | 292 |
| 61 | digital thermometer*.tw. | 9 |
| 62 | "thermal camera*".tw. | 9 |
| 63 | exp Virtual Reality/ | 8701 |
| 64 | "virtual reality".tw. | 6079 |
| 65 | exp Augmented Reality/ | 484 |
| 66 | "augmented reality".tw. | 779 |
| 67 | "contact tracing tool*".tw. | 0 |
| 68 | "contact tracing app*".tw. | 0 |
| 69 | "contact tracing device*".tw. | 0 |
| 70 | chatbot*.tw. | 79 |
| 71 | "chat-bot*".tw. | 16 |
| 72 | "chat bot*".tw. | 16 |
| 73 | "dialogue agent*".tw. | 3 |
| 74 | "conversational agent*".tw. | 224 |
| 75 | drone*.tw. | 269 |
| 76 | "computer vision".tw. | 952 |
| 77 | exp Text Messaging/ | 974 |
| 78 | "instant messag*".tw. | 732 |
| 79 | "text messag*".tw. | 2352 |
| 80 | exp Social Media/ | 14648 |
| 81 | "social media".tw. | 12604 |
| 82 | Twitter.tw. | 2833 |
| 83 | Facebook.tw. | 5449 |
| 84 | Instagram.tw. | 530 |
| 85 | Youtube.tw. | 1193 |
| 86 | Whatsapp.tw. | 159 |
| 87 | Wechat.tw. | 108 |
| 88 | "Tik ToK".tw. | 0 |
| 89 | Snapchat.tw. | 121 |
| 90 | "novel coronavirus".tw. | 95 |
| 91 | "coronavirus 2019".tw. | 24 |
| 92 | "novel corona virus".tw. | 2 |
| 93 | "corona virus 2019".tw. | 0 |
| 94 | "COVID 2019".tw. | 4 |
| 95 | "COVID 19".tw. | 978 |
| 96 | "COVID19".tw. | 7 |
| 97 | "COVID-19".tw. | 978 |
| 98 | "SARS-CoV-2".tw. | 50 |
| 99 | "HCoV-19".tw. | 0 |
| 100 | "2019-nCoV".tw. | 13 |
| 101 | "severe acute respiratory syndrome coronavirus 2".tw. | 18 |
| 102 | "Wuhan coronavirus".tw. | 0 |
| 103 | "Wuhan corona virus".tw. | 0 |
| 104 | "Chinese coronavirus".tw. | 0 |
| 105 | 1 or 2 or 3 or 4 or 5 or 6 or 7 or 8 or 9 or 10 or 11 or 12 or 13 or 14 or 15 or 16 or 17 or 18 or 19 or 20 or 21 or 22 or 23 or 24 or 25 or 26 or 27 or 28 or 29 or 30 or 31 or 32 or 33 or 34 or 35 or 36 or 37 or 38 or 39 or 40 or 41 or 42 or 43 or 44 or 45 or 46 or 47 or 48 or 49 or 50 or 51 or 52 or 53 or 54 or 55 or 56 or 57 or 58 or 59 or 60 or 61 or 62 or 63 or 64 or 65 or 66 or 67 or 68 or 69 or 70 or 71 or 72 or 73 or 74 or 75 or 76 or 77 or 78 or 79 or 80 or 81 or 82 or 83 or 84 or 85 or 86 or 87 or 88 or 89 | 188214 |
| 106 | 90 or 91 or 92 or 93 or 94 or 95 or 96 or 97 or 98 or 99 or 100 or 101 or 102 or 103 or 104 | 995 |
| 107 | 105 and 106 | 174 |
| 108 | limit 107 to yr="2020 -Current" | 174 |
| 109 | limit 108 to english language | 84 |

Database(s): **Scopus**

| **#** | **Search Terms** | **Results** |
| --- | --- | --- |
| 1 | TITLE-ABS-KEY ( technolog*  OR  "e-health"  OR  ehealth  OR  "digital health"  OR  telemedicine  OR  "tele-medicine"  OR  telehealth  OR  "tele-health"  OR  telecare  OR  "tele-care"  OR  telepsychiatr*  OR  "tele-psychiatr*"  OR  teledermatology  OR  "tele-dermatology" ) | 3,654,692 |
| 2 | TITLE-ABS-KEY ( teleconsultation  OR  "tele-consultation"  OR  teleconferenc*  OR  "tele-conferenc*"  OR  telesurger*  OR  "tele-surger*"  OR  "electronic prescribing"  OR  "e-prescribing"  OR  "computerized provider order entry"  OR  "computerised provider order entry"  OR  cpoe ) | 22,063 |
| 3 | TITLE-ABS-KEY ( "patient portal*"  OR  "personal health record*"  OR  "electronic health record*"  OR  "electronic medical record*"  OR  "kiosk*"  OR  "decision support system*"  OR  "mobile health"  OR  "m-health"  OR  mhealth  OR  "mobile app*"  OR  "mobile phone app*"  OR  "smartphone app*" ) | 229,679 |
| 4 | TITLE-ABS-KEY ( "smart phone app*"  OR  "artificial intelligence"  OR  "machine learning"  OR  "deep learning"  OR  "neural network"  OR  wearable*  OR  "sensor*"  OR  "digital tool*"  OR  "digital device*"  OR  robot*  OR  dashboard*  OR  "digital thermometer*"  OR  "thermal camera*" ) | 2,944,781 |
| 5 | TITLE-ABS-KEY ( "virtual reality"  OR  "augmented reality"  OR  "contact tracing app*"  OR  "contact tracing tool*"  OR  "contact tracing device*"  OR  chatbot*  OR  "chat-bot*"  OR  "chat bot*"  OR  "dialogue agent*"  OR  "conversational agent*" ) | 147,095 |
| 6 | TITLE-ABS-KEY ( drone*  OR  "computer vision"  OR  "instant messag*"  OR  "text messag*"  OR  "social media"  OR  twitter  OR  facebook  OR  instagram  OR  youtube  OR  whatsapp  OR  wechat  OR  "Tik ToK" ) | 286,499 |
| 7 | TITLE-ABS-KEY ( "novel coronavirus"  OR  "coronavirus 2019"  OR  "novel corona virus"  OR  "corona virus 2019"  OR  "COVID 2019"  OR  "COVID19"  OR  "COVID 19"  OR  "COVID-19"  OR  "SARS-CoV-2"  OR  "HCoV-19"  OR  "2019-nCoV"  OR  "severe acute respiratory syndrome coronavirus 2" ) | 39,184 |
| 8 | TITLE-ABS-KEY ( "Wuhan coronavirus"  OR  "Wuhan corona virus" ) | 23 |
| 9 | (#1 OR #2 OR #3 OR #4 OR #5 OR #6) AND (#7 OR #8) | 4,560 |
| 10 | #9 AND ( LIMIT-TO ( DOCTYPE ,  "ar" )  OR  LIMIT-TO ( DOCTYPE ,  "cp" )  OR  LIMIT-TO ( DOCTYPE ,  "ch" ) )  AND  ( LIMIT-TO ( LANGUAGE ,  "English" ) )  AND  ( LIMIT-TO ( SRCTYPE ,  "j" )  OR  LIMIT-TO ( SRCTYPE ,  "p" ) ) | 2,490 |

| **Database** | **Search Terms** | **Results** |
| --- | --- | --- |
| ACM library | [Abstract: "novel coronavirus"] OR [Abstract: "coronavirus 2019"] OR [Abstract: "novel corona virus"] OR [Abstract: "corona virus 2019"] OR [Abstract: "covid 2019"] OR [Abstract: "covid19"] OR [Abstract: "covid 19"] OR [Abstract: "covid-19"] OR [Abstract: "sars-cov-2"] OR [Abstract: "hcov-19"] OR [Abstract: "2019-ncov"] OR [Abstract: "severe acute respiratory syndrome coronavirus 2"] OR [Abstract: "wuhan coronavirus"] OR [Abstract: "wuhan corona virus"] AND [Publication Date: (01/01/2020 TO 12/31/2020)] | 89 |
| Web of science | (Technolog*  OR "e-health"  OR ehealth  OR "digital health"  OR telemedicine  OR "tele-medicine"  OR telehealth  OR "tele-health"  OR telecare  OR "tele-care"  OR telepsychiatr*  OR "tele-psychiatr*"  OR teledermatology  OR "tele-dermatology"  OR teleconsultation  OR "tele-consultation"  OR teleconferenc*  OR "tele-conferenc*"  OR telesurger*  OR "tele-surger*"  OR "electronic prescribing"  OR "e-prescribing"  OR "computerized provider order entry"  OR "computerised provider order entry"  OR CPOE  OR "patient portal*"  OR "personal health record*"  OR "electronic health record*"  OR "electronic medical record*"  OR "kiosk*"  OR "decision support system*"  OR "mobile health"  OR "m-health"  OR mhealth  OR "mobile app*"  OR "mobile phone app*"  OR "smartphone app*"  OR "smart phone app*"  OR "artificial intelligence"  OR "machine learning"  OR "deep learning"  OR "neural network"  OR wearable*  OR "sensor*"  OR "digital tool*"  OR "digital device*"  OR robot*  OR dashboard*  OR "digital thermometer*"  OR "thermal camera*"  OR "virtual reality"  OR "augmented reality"  OR "contact tracing app*"  OR "contact tracing tool*"  OR "contact tracing device*"  OR chatbot*  OR "chat-bot*"  OR "chat bot*"  OR "dialogue agent*"  OR "conversational agent*"  OR drone*  OR "computer vision"  OR "instant messag*"  OR "text messag*"  OR "social media"  OR Twitter  OR Facebook  OR Instagram  OR Youtube  OR Whatsapp  OR Wechat  OR “Tik ToK”) AND TOPIC: ("novel coronavirus"  OR "coronavirus 2019"  OR "novel corona virus"  OR "corona virus 2019"  OR "COVID 2019"  OR "COVID19"  OR "COVID 19"  OR "COVID-19"  OR "SARS-CoV-2"  OR "HCoV-19"  OR "2019-nCoV"  OR "severe acute respiratory syndrome coronavirus 2"  OR "Wuhan coronavirus"  OR "Wuhan corona virus")  Refined by: DOCUMENT TYPES: ( ARTICLE OR EARLY ACCESS OR PROCEEDINGS PAPER ) AND LANGUAGES: ( ENGLISH )  Timespan: 2020. Indexes: SCI-EXPANDED, SSCI, A&HCI, CPCI-S, CPCI-SSH, BKCI-S, BKCI-SSH, ESCI, CCR-EXPANDED, IC. | 1430 |
| IEEE Xplore | ("Abstract":"novel coronavirus" OR "coronavirus 2019" OR "novel corona virus" OR "corona virus 2019" OR "COVID 2019" OR "COVID19" OR "COVID 19" OR "COVID-19" OR "SARS-CoV-2" OR "HCoV-19" OR "2019-nCoV" OR "severe acute respiratory syndrome coronavirus 2" OR "Wuhan coronavirus" OR "Wuhan corona virus") | 251 |
| Google Scholar | ("COVID 2019" OR "COVID19" OR "COVID-19" OR “novel coronavirus") AND (“technolog* OR "e-health" OR "digital health" OR telemedicine) | 100 |
