# Supplementary material for: Overview of Technologies Implemented During the First Wave of the COVID-19 Pandemic: Scoping Review
Source: J Med Internet Res. 2021 Sep 14;23(9):e29136. doi: 10.2196/29136 (PMC8767979; doi:10.2196/29136)
Supplement: Multimedia Appendix 2 [file jmir_v23i9e29136_app2.docx]

| **Concept** | **Definition** |
| --- | --- |
| **Study Characteristics** |  |
| Author | The first author of the study. |
| Month of submission | The month in which the study was submitted. |
| Country of publication | The country where the study was published. |
| Type of publication | The medium in which the review was published (e.g. conference proceedings, peer-reviewed journal, thesis) |
| **Technology characteristics** |  |
| Technology type | To which group technology belongs to (e.g., telemedicine, clinical decision support tools, symptom trackers, dashboards, triage tools, etc.) |
| Mode of telemedicine | Was it live interactive telemedicine (synchronous) or store-and-forward telemedicine (asynchronous)? |
| Technology aim | What is the purpose of implementing the technology? |
| Technology development | Was the technology was designed to provide the respective purpose from the beginning (Built for purpose) or was used for other purposes rather than the purpose that they were developed for (Purpose-shifted) |
| Social media and video-conferencing platforms | What are social media and video-conferencing platforms used in the papers (e.g., WhatsApp, Zoom, WebEx) |
| Target users | Who are the users of the technology |
| Target conditions | What is the health condition that the technology endeavor to improve |
| Setting | To which setting the users belong to (e.g., hospitals, Medical center, educational settings, etc.) |
| Internet connectivity | Is the technology based on internet? |
| Venues | What is the venue that technology is used through |

**Appendix 2: Data extraction form**
