# Supplementary material for: Overview of Technologies Implemented During the First Wave of the COVID-19 Pandemic: Scoping Review
Source: J Med Internet Res. 2021 Sep 14;23(9):e29136. doi: 10.2196/29136 (PMC8767979; doi:10.2196/29136)
Supplement: Multimedia Appendix 3 [file jmir_v23i9e29136_app3.docx]

**Appendix 3: Characteristics of each included study.**

| Author^ID^ | Submission month | Country | Paper status |
| --- | --- | --- | --- |
| Alromaihi^10^ | June | Bahrain | Journal article |
| Li, L.^11^ | August | China | Journal article |
| Liu, L.^12^ | July | China | Journal article |
| Lonergan^13^ | July | USA | Journal article |
| Mader^14^ | May | Austria | Journal article |
| Mann^15^ | May | USA | Journal article |
| Marasca^16^ | August | Italy | Journal article |
| McGinley^17^ | June | USA | Journal article |
| Nørgaard^18^ | May | Denmark | Journal article |
| Opinc^19^ | June | Poland | Journal article |
| Panda^20^ | July | India | Journal article |
| Pandey^21^ | June | India | Journal article |
| Peden^22^ | June | USA | Journal article |
| Perez-Alba^23^ | April | Mexico | Journal article |
| Perniola^24^ | June | Italy | Journal article |
| Poulose^25^ | June | India | Journal article |
| Rabunal^26^ | June | Spain | Journal article |
| Rametta^27^ | May | USA | Journal article |
| Ramtekkar^28^ | June | USA | Journal article |
| Rodler^29^ | June | Germany | Journal article |
| Roncero^30^ | July | Spain | Journal article |
| Saccomanno^31^ | June | Italy | Journal article |
| Schulz^32^ | July | Australia | Journal article |
| Serper^33^ | June | USA | Journal article |
| Shelton^34^ | June | USA | Journal article |
| Shenoy^35^ | May | India | Journal article |
| Siow^36^ | July | USA | Journal article |
| Wolthers^37^ | June | Denmark | Journal article |
| Aziz^38^ | April | USA | Journal article |
| Geoffroy ^39^ | May | Canada | Journal article |
| Gilbert^40^ | May | UK | Journal article |
| Ren^41^ | June | China | Journal article |
| Sossai^42^ | July | Italy | Journal article |
| DiGiovanni^43^ | June | USA | Journal article |
| Dosaj^44^ | July | USA | Journal article |
| Margusino^45^ | June | Spain | Journal article |
| McElroy^46^ | July | USA | Journal article |
| Datta^47^ | June | USA | Journal article |
| Zhao^48^ | May | China | Journal article |
| Song^49^ | April | China | Journal article |
| Das^50^ | May | India | Journal article |
| Di Tommaso^51^ | April | Argentina | Journal article |
| Luciani^52^ | April | Italy | Journal article |
| Madden^53^ | June | USA | Journal article |
| Mostafa^54^ | July | Egypt | Journal article |
| Mouchtouris^55^ | June | USA | Journal article |
| Nakagawa^56^ | July | Japan | Journal article |
| Pagliazzi^57^ | August | Italy | Journal article |
| Pluymaekers^58^ | May | Australia | Journal article |
| Prada^59^ | April | Italy | Journal article |
| Rastogi^60^ | May | India | Journal article |
| Rismiller^61^ | May | India | Journal article |
| Salzano^62^ | May | Italy | Journal article |
| Smrke^63^ | July | UK | Journal article |
| Strohl^64^ | June | USA | Journal article |
| Swierad^65^ | May | Poland | Journal article |
| Tanaka^66^ | April | USA | Journal article |
| Tenforde^67^ | May | USA | Journal article |
| Wang, Y.^68^ | July | China | Journal article |
| Watts^69^ | June | USA | Journal article |
| Wosik^70^ | May | USA | Journal article |
| Yellowlees^71^ | July | USA | Journal article |
| Zweig^72^ | May | USA | Report |
| Sullivan^73^ | May | USA | Journal article |
| Lee^74^ | April | China | Journal article |
| Krausz^75^ | June | Canada | Journal article |
| Lau^76^ | June | USA | Journal article |
| Sampa^77^ | June | Japan | Journal article |
| Liu, Y.^78^ | May | China | Journal article |
| Paleri^79^ | May | UK | Journal article |
| Milenkovic^80^ | June | Serbia | Journal article |
| Ayoub^81^ | June | USA | Journal article |
| Harris^82^ | July | USA | Journal article |
| Linz^83^ | May | Netherlands | Journal article |
| Barney^84^ | May | USA | Journal article |
| Obeid^85^ | July | USA | Journal article |
| Lin, C.^86^ | June | Taiwan | Journal article |
| Pignatti^87^ | May | Italy | Journal article |
| Agyapong^88^ | April | Canada | Journal article |
| Yadav^89^ | August | USA | Journal article |
| Salway^90^ | July | USA | Journal article |
| Timmers^91^ | June | Netherlands | Journal article |
| Lam^92^ | May | Canada | Journal article |
| Li, H.^93^ | June | China | Journal article |
| Lin, J. ^94^ | July | USA | Journal article |
| Ma^95^ | February | China | Journal article |
| Meloni^96^ | June | Italy | Journal article |
| Palomba^97^ | May | Italy | Journal article |
| Punia^98^ | May | India | Journal article |
| Qualliotine^99^ | April | USA | Journal article |
| Naik100 | June | India | Journal article |
| Thornton^101^ | June | UK | Journal article |
| Umoren^102^ | April | USA | Journal article |
| Martinez^103^ | July | Spain | Journal article |
| Xu^104^ | July | China | Journal article |
| Vilendrer^105^ | June | USA | Journal article |
| Ratwani^106^ | July | USA | Journal article |
| McKiever^107^ | August | USA | Journal article |
| Medalia^108^ | August | USA | Report |
| Miu^109^ | July | USA | Journal article |
| Sequeira^110^ | June | USA | Journal article |
| Stewart^111^ | April | USA | Journal article |
| Sharma^112^ | August | USA | Journal article |
| Dimer^113^ | May | Brazil | Journal article |
| Reeves^114^ | April | USA | Journal article |
| Low^115^ | August | Singapore | Journal article |
| Martin^116^ | August | UK | Journal article |
| Nagaratnam^117^ | June | UK | Journal article |
| Shamout^118^ | August | USA | Preprint |
| Gong^119^ | April | China | Journal article |
| Miseikis^120^ | July | Germany | Journal article |
| Rane^121^ | June | India | Journal article |
| Tabaza^122^ | May | USA | Journal article |
| Wang, J. ^123^ | July | USA | Journal article |
| Ye^124^ | August | China | Journal article |
| Yu^125^ | July | China | Journal article |
| Drew^126^ | May | USA | Journal article |
| Menni1^27^ | May | UK | Journal article |
| Yamamoto^128^ | July | Japan | Journal article |
| Schinkothe^129^ | June | Germany | Journal article |
| Dixit^130^ | July | USA | Journal article |
| Stevens^131^ | July | USA | Journal article |
| Ntshalintshali^132^ | July | South Africa | Journal article |
| Lai^133^ | July | USA | Journal article |
| Li, C.^134^ | June | USA | Journal article |
| Li, J.^135^ | May | China | Journal article |
