# Supplementary material for: Overview of Technologies Implemented During the First Wave of the COVID-19 Pandemic: Scoping Review
Source: J Med Internet Res. 2021 Sep 14;23(9):e29136. doi: 10.2196/29136 (PMC8767979; doi:10.2196/29136)
Supplement: Multimedia Appendix 4 [file jmir_v23i9e29136_app4.docx]

**Appendix 4: Characteristics of technologies in each included study.**

| Author^ID^ | Technology type | Technology aim | Mode of telemedicine | Social media & video-conferencing platforms | Technology development | Target users | Target conditions | Setting | Internet connectivity | Venues |
| --- | --- | --- | --- | --- | --- | --- | --- | --- | --- | --- |
| Alromaihi^10^ | Telemedicine | Consultation | Synchronous | Unspecified | Unspecified | Health consumers & Healthcare professionals | Diabetes | Hospital | Both | Mobile app, Telephone call |
| Li, L.^11^ | Telemedicine | Consultation | Synchronous | WeChat | Purpose-shifted | Health consumers & Healthcare professionals | Cardiovascular disorders | Hospital | Web-based | Mobile App, Desktop app, Website |
| Liu, L.^12^ | Telemedicine | Consultation | Synchronous | WeChat | Purpose-shifted | Health consumers & Healthcare professionals | General | Hospital | Web-based | Mobile App, Desktop app, Website |
| Lonergan^13^ | Telemedicine | Consultation | Synchronous | Zoom | Purpose-shifted | Health consumers & Healthcare professionals | Cancer | Medical center | Web-based | Mobile App, Desktop app, Website |
| Mader^14^ | Telemedicine | Consultation | Synchronous | Unspecified | Unspecified | Health consumers & Healthcare professionals | Diabetes | Hospital | Non-web-based | Telephone call |
| Mann^15^ | Telemedicine | Consultation | Synchronous | MyChart | Purpose-shifted | Health consumers & Healthcare professionals | General | Hospital | Web-based | Mobile App, Website |
| Marasca^16^ | Telemedicine | Consultation | Synchronous | Unspecified | Unspecified | Health consumers & Healthcare professionals | Dermatological conditions | Medical center | Web-based | Mobile app, Desktop app |
| McGinley^17^ | Telemedicine | Consultation | Synchronous | FaceTime, Google Duo | Both | Health consumers & Healthcare professionals | Neurological disorders | Medical center | Both | Mobile App, Desktop app, Website, Telephone call |
| Nørgaard^18^ | Telemedicine | Consultation | Synchronous | Unspecified | Unspecified | Health consumers & Healthcare professionals | Diabetes | Hospital | Non-web-based | Telephone call |
| Opinc^19^ | Telemedicine | Consultation | Synchronous | Unspecified | Unspecified | Health consumers & Healthcare professionals | Rheumatic disorders | Hospital | Non-web-based | Telephone call |
| Panda^20^ | Telemedicine | Consultation | Asynchronous | WhatsApp | Purpose-shifted | Health consumers & Healthcare professionals | Neurological disorders | Hospital | Both | Mobile App, Desktop app, Website, Telephone call |
| Pandey^21^ | Telemedicine | Consultation | Asynchronous | WhatsApp | Purpose-shifted | Health consumers & Healthcare professionals | Opthalmic illnesses | Medical center | Both | Mobile App, Desktop app, Website, Telephone call |
| Peden^22^ | Telemedicine | Consultation | Synchronous | InTouch Health, Google Duo, Messenger, Telegram, WhatsApp, Zoom | Both | Health consumers & Healthcare professionals | General | Medical center | Web-based | Mobile App, Desktop app, Website |
| Perez-Alba^23^ | Telemedicine | Consultation | Synchronous | GoogleForms, Skype | Purpose-shifted | Health consumers & Healthcare professionals | COVID-19 | Medical center | Both | Mobile App, Desktop app, Website, Interphone |
| Perniola^24^ | Telemedicine | Consultation | Both | Unspecified | Unspecified | Health consumers & Healthcare professionals | Rheumatic disorders | Hospital | Both | Telephone call, Email |
| Poulose^25^ | Telemedicine | Consultation | Synchronous | Unspecified | Unspecified | Health consumers & Healthcare professionals | General | Hospital | Both | Mobile app, Desktop app, Telephone call |
| Rabunal^26^ | Telemedicine | Consultation | Both | TELEA | Built for purpose | Health consumers & Healthcare professionals | COVID-19 | Hospital | Both | Desktop app, Telephone call, Email |
| Rametta^27^ | Telemedicine | Consultation | Synchronous | Unspecified | Unspecified | Health consumers & Healthcare professionals | Neurological disorders | Hospital | Both | Mobile app, Desktop app, Telephone call |
| Ramtekkar^28^ | Telemedicine | Consultation | Synchronous | Zoom | Purpose-shifted | Health consumers & Healthcare professionals | Mental illnesses | Hospital | Both | Mobile App, Desktop app, Website, Telephone call |
| Rodler^29^ | Telemedicine | Consultation | Both | Unspecified | Unspecified | Health consumers & Healthcare professionals | Cancer | Hospital | Both | Mobile app, Desktop app, Telephone call, Email |
| Roncero^30^ | Telemedicine | Consultation | Synchronous | Unspecified | Unspecified | Healthcare Professionals | Mental illnesses | Hospital | Non-web-based | Telephone call |
| Saccomanno^31^ | Telemedicine | Consultation | Synchronous | Smile consult, Zoom | Both | Health consumers & Healthcare professionals | Orthodontic issues | Hospital | Web-based | Mobile App, Desktop app, Website |
| Schulz^32^ | Telemedicine | Consultation | Both | Unspecified | Unspecified | Health consumers & Healthcare professionals | General | Hospital | Both | Desktop app, Telephone call, Email |
| Serper^33^ | Telemedicine | Consultation | Synchronous | Unspecified | Unspecified | Health consumers & Healthcare professionals | Gastrointestinal illness, Liver illness | Medical center | Web-based | Mobile App, Desktop app |
| Shelton^34^ | Telemedicine | Consultation | Synchronous | Zoom | Purpose-shifted | Health consumers & Healthcare professionals | Diabetes | Hospital | Web-based | Mobile App, Desktop app, Website |
| Shenoy^35^ | Telemedicine | Consultation | Synchronous | WhatsApp | Purpose-shifted | Health consumers & Healthcare professionals | Rheumatic disorders | Medical center | Web-based | Mobile App, Desktop app, Website |
| Siow^36^ | Telemedicine | Consultation | Synchronous | MyChart | Purpose-shifted | Health consumers & Healthcare professionals | Orthopaedic conditions | Medical center | Web-based | Mobile App, Website |
| Wolthers^37^ | Telemedicine | Consultation | Synchronous | Unspecified | Unspecified | Health consumers & Healthcare professionals | General | Medical center | Non-web-based | Telephone call |
| Aziz^38^ | Telemedicine | Consultation | Synchronous | EpicConnect, MyChart | Built for purpose | Health consumers & Healthcare professionals | Prenatal and postnatal conditions | Medical center, Hospital | Web-based | Mobile App, Website |
| Geoffroy ^39^ | Telemedicine | Consultation | Synchronous | Unspecified | Unspecified | Healthcare professionals | Mental illnesses | Hospital | Non-web-based | Telephone call |
| Gilbert^40^ | Telemedicine | Consultation | Synchronous | Unspecified | Unspecified | Health consumers & Healthcare professionals | Orthopaedic conditions | Hospital | Both | Mobile app, Telephone call |
| Ren^41^ | Telemedicine, EHR | Consultation, Accessing patient records | Synchronous | Unspecified | Built for purpose | Healthcare Professionals | COVID-19 | Hospital | Web-based | Mobile App |
| Sossai^42^ | Telemedicine | Consultation, Diagnosis | Synchronous | DaVinci Salute | Built for purpose | Health consumers & Healthcare professionals | COVID-19 | Medical center | Web-based | Mobile App |
| DiGiovanni^43^ | Telemedicine | Consultation, Diagnosis, Education | Both | Unspecified | Unspecified | Health consumers & Healthcare professionals | Cancer | Medical center | Both | Telephone call, Email |
| Dosaj^44^ | Telemedicine | Consultation, Diagnosis, Education, Treatment | Synchronous | InTouch Health, WebEx | Both | Health consumers & Healthcare professionals | Prenatal and postnatal conditions | Medical center | Web-based | Mobile App, Desktop app, Website |
| Margusino^45^ | Telemedicine | Consultation, Drug delivery | Asynchronous | Unspecified | Unspecified | Health consumers & Healthcare professionals | General | Hospital | Web-based | Text messages |
| McElroy^46^ | Telemedicine | Consultation, Education | Synchronous | Unspecified | Built for purpose | Health consumers & Healthcare professionals | General | Medical center | Both | Mobile App, Telephone call |
| Datta^47^ | Telemedicine | Consultation, Education, Follow up | Synchronous | Zoom | Purpose-shifted | Health consumers & Healthcare professionals | Mental illnesses | Hospital | Web-based | Mobile App, Desktop app, Website |
| Zhao^48^ | Telemedicine | Consultation, Education, Follow up | Synchronous | Cloud Clinic | Built for purpose | Health consumers & Healthcare professionals | Transplant patients | Hospital | Web-based | Website |
| Song^49^ | Telemedicine | Consultation, Education, Follow up, Triage | Both | Unspecified | Unspecified | Health consumers & Healthcare professionals | COVID-19 | Medical center, Hospital | Both | Desktop app, Telephone call |
| Das^50^ | Telemedicine | Consultation, Follow up | Both | GoogleForms, WhatsApp | Purpose-shifted | Health consumers & Healthcare professionals | Opthalmic illnesses | Hospital | Both | Mobile app, Desktop app, Website, Telephone call, Email |
| Di Tommaso^51^ | Telemedicine | Consultation, Follow up | Synchronous | WhatsApp | Purpose-shifted | Health consumers & Healthcare professionals | General | Hospital | Web-based | Mobile App, Desktop app, Website |
| Luciani^52^ | Telemedicine | Consultation, Follow up | Synchronous | Unspecified | Unspecified | Health consumers & Healthcare professionals | General | Hospital | Non-web-based | Telephone call |
| Madden^53^ | Telemedicine | Consultation, Follow up | Synchronous | EpicConnect, MyChart | Built for purpose | Health consumers & Healthcare professionals | Prenatal and postnatal conditions | Hospital | Web-based | Mobile App, Website |
| Mostafa^54^ | Telemedicine | Consultation, Follow up | Synchronous | WhatsApp, Zoom, Emails | Purpose-shifted | Health consumers & Healthcare professionals | Dermatological conditions | Medical center, Hospital | Web-based | Mobile App, Desktop app, Website, Email |
| Mouchtouris^55^ | Telemedicine | Consultation, Follow up | Synchronous | Teladoc Health,VidyoConnect | Built for purpose | Health consumers & Healthcare professionals | Neurological disorders | Hospital | Web-based | Desktop app |
| Nakagawa^56^ | Telemedicine | Consultation, Follow up | Synchronous | Unspecified | Unspecified | Health consumers & Healthcare professionals | Prenatal and postnatal conditions | Hospital | Non-web-based | Telephone call |
| Pagliazzi^57^ | Telemedicine | Consultation, Follow up | Both | Unspecified | Unspecified | Health consumers & Healthcare professionals | Prenatal and postnatal conditions | Hospital | Both | Telephone call, Email |
| Pluymaekers^58^ | Telemedicine | Consultation, Follow up | Both | FibriCheck | Built for purpose | Health consumers | Cardiovascular disorders | Medical center | Both | Mobile App, Telephone call |
| Prada^59^ | Telemedicine | Consultation, Follow up | Synchronous | Google Duo, Telegram, Skype, WhatsApp, Zoom | Purpose-shifted | Health consumers & Healthcare professionals | Neurological disorders | Medical center | Both | Mobile App, Desktop app, Website, Telephone call |
| Rastogi^60^ | Telemedicine | Consultation, Follow up | Synchronous | WhatsApp | Purpose-shifted | Health consumers & Healthcare professionals | Rheumatic disorders | Medical center | Web-based | Mobile App, Desktop app, Website |
| Rismiller^61^ | Telemedicine | Consultation, Follow up | Synchronous | WebEx | Purpose-shifted | Health consumers & Healthcare professionals | Dermatological conditions | Hospital | Web-based | Mobile App, Desktop app, Website |
| Salzano^62^ | Telemedicine | Consultation, Follow up | Both | Unspecified | Unspecified | Health consumers & Healthcare professionals | Cardiovascular disorders | Medical center | Both | Mobile app, Desktop app, Telephone call, Email |
| Smrke^63^ | Telemedicine | Consultation, Follow up | Synchronous | Unspecified | Unspecified | Health consumers & Healthcare professionals | Cancer | Hospital | Non-web-based | Telephone call |
| Strohl^64^ | Telemedicine | Consultation, Follow up | Synchronous | Zoom | Purpose-shifted | Health consumers & Healthcare professionals | ENT issues | Medical center | Both | Mobile App, Desktop app, Website, Telephone call |
| Swierad^65^ | Telemedicine | Consultation, Follow up | Synchronous | Unspecified | Unspecified | Health consumers & Healthcare professionals | Cardiovascular disorders | Medical center | Non-web-based | Telephone call |
| Tanaka^66^ | Telemedicine | Consultation, Follow up | Synchronous | Doxy.me, InTouch Health, Zoom | Both | Health consumers & Healthcare professionals | Orthopaedic conditions | Hospital | web-based | Mobile App, Desktop app, Website |
| Tenforde^67^ | Telemedicine | Consultation, Follow up | Synchronous | InTouch Health, Zoom | Both | Health consumers & Healthcare professionals | Orthopaedic conditions | Hospital | Web-based | Mobile App, Desktop app, Website |
| Wang, Y.^68^ | Telemedicine | Consultation, Follow up | Synchronous | WeChat | Purpose-shifted | Health consumers & Healthcare professionals | Opthalmic illnesses | Hospital | web-based | Mobile App, Desktop app, Website |
| Watts^69^ | Telemedicine | Consultation, Follow up | Synchronous | FaceTime, Zoom | Purpose-shifted | Health consumers & Healthcare professionals | Urologic diseases | Hospital | web-based | Mobile App, Desktop app, Website |
| Wosik^70^ | Telemedicine | Consultation, Follow up | Synchronous | ExtendedCare (Outpatient) & TeleICU (Inpatient) | Built for purpose | Health consumers & Healthcare professionals | General | Hospital | Web-based | Mobile app, Desktop app |
| Yellowlees^71^ | Telemedicine | Consultation, Follow up | Synchronous | MyChart, Zoom | Purpose-shifted | Health consumers & Healthcare professionals | Mental illnesses | Medical center | Both | Mobile App, Desktop app, Website |
| Zweig^72^ | Telemedicine | Consultation, Follow up | Synchronous | Zoom | Purpose-shifted | Health consumers & Healthcare professionals | COVID-19 | Medical center | Web-based | Mobile App, Desktop app, Website |
| Sullivan^73^ | Telemedicine, Patinet portal | Consultation, Follow up | Synchronous | Facetime, Google Duo, MyChart, Skype, Zoom | Both | Health consumers & Healthcare professionals | Mental illnesses | medical center | Both | Mobile App, Desktop app, Website, Telephone call |
| Lee^74^ | Telemedicine | Consultation, Follow up, Education | Both | Zoom | Purpose-shifted | Health consumers & Healthcare professionals | Cancer | Hospital | Both | Mobile App, Desktop app, Website, Telephone call, Email |
| Krausz^75^ | Telemedicine, Dashboard, CDSS, Symptom tracker | Consultation, Follow up, Monitoring (patients), Triage, Reporting, Booking appointments | Synchronous | Unspecified | Unspecified | Health consumers & Healthcare professionals | COVID-19 | Community | Web-based | Mobile App, Desktop app |
| Lau^76^ | Telemedicine | Consultation, Follow up, Treatment, Triage | Synchronous | Unspecified | Unspecified | Health consumers & Healthcare professionals | General | Hospital | Both | Telephone call |
| Sampa^77^ | Telemedicine, CDSS | Consultation, Follow up, Screening | Both | GramHealth | Built for purpose | Health consumers & Healthcare professionals | General | Hospital | Web-based | Mobile app, Desktop app |
| Liu, Y.^78^ | Telemedicine, CDSS | Consultation, Follow up, Screening | Asynchronous | DDC19 | Built for purpose | Health consumers & Healthcare professionals | COVID-19 | Medical center | Both | Mobile App |
| Paleri^79^ | Telemedicine, CDSS | Consultation, Triage | Synchronous | Unspecified | Unspecified | Health consumers & Healthcare professionals | Cancer | Hospital | Non-web-based | Desktop app, Telephone call |
| Milenkovic^80^ | Telemedicine, CDSS (AI-based), Patient portal, Reporting system | Consultation, Triage, Schedualing, Medical data exchange, Reporting | Synchronous | MEDIS.NE | Built for purpose | Health consumers & Healthcare professionals | General | Medical center | Web-based | Desktop app, Website |
| Ayoub^81^ | Telemedicine | Consultation, Triage | Synchronous | Unspecified | Unspecified | Health consumers & Healthcare professionals | General | Medical center | Both | Mobile app, Telephone call |
| Harris^82^ | Telemedicine | Consultation, Monitoring (patients), Treatment | Synchronous | Unspecified | Unspecified | Healthcare professionals | COVID-19 | Hospital | Web-based | Mobile app, Desktop app |
| Linz^83^ | Telemedicine | Consultation, Monitoring (patients), Treatment | Synchronous | TeleCheck-AF | Built for purpose | Health consumers & Healthcare professionals | Cardiovascular disorders | Medical center | Web-based | Mobile App |
| Barney^84^ | Telemedicine | Consultation, Monitoring (patients), Triage | Synchronous | Zoom | Purpose-shifted | Health consumers & Healthcare professionals | Mental illnesses, Reproductive disorders | Medical center | web-based | Mobile App, Desktop app, Website |
| Obeid^85^ | Telemedicine, CDSS | Triage | Synchronous | Unspecified | Unspecified | Health consumers & Healthcare professionals | COVID-19 | Medical center, Hospital | Both | Mobile app, Desktop app |
| Lin, C.^86^ | Telemedicine, CDSS | Triage, Diagnosis | Synchronous | TTAS, U Meeting | Built for purpose | Health consumers & Healthcare professionals | COVID-19 | Hospital | Both | Desktop app |
| Pignatti^87^ | Telemedicine | Triage, Follow up | Both | Messenger, Telegram, WhatsApp, Emails | Both | Health consumers & Healthcare professionals | General | Hospital | Both | Mobile App, Desktop app, Website, Telephone call, Email |
| Agyapong^88^ | Telemedicine | Education | Asynchronous | Text4Hope | Built for purpose | Health consumers & Healthcare professionals | Mental illnesses | Community | Non-web-based | Text messages |
| Yadav^89^ | Telemedicine | Education, Clinical assessment | Synchronous | Unspecified | Unspecified | Health consumers & Healthcare professionals | Urologic diseases | Hospital | Both | Mobile app, Desktop app, Telephone call |
| Salway^90^ | Telemedicine, Dashboard, Triage tool | Education, Monitoring (patients & services), Triage, Connecting patients and families | Synchronous | SmartNotes | Built for purpose | Health consumers & Healthcare professionals | COVID-19 | Medical center, Hospital | Both | Mobile app, Desktop app, Vital-sign monitor |
| Timmers^91^ | Telemedicine | Education, Monitoring (patients) | Asynchronous | ETZ app | Built for purpose | Health consumers | COVID-19 | hospital | Web-based | Mobile App |
| Lam^92^ | Telemedicine | Follow up | Both | COVIDEO | Built for purpose | Health consumers & Healthcare professionals | COVID-19 | Medical center | Both | Website, Telephone call, Email |
| Li, H.^93^ | Telemedicine | Follow up | Synchronous | WeChat | Purpose-shifted | Health consumers & Healthcare professionals | Cardiovascular disorders | Hospital | Web-based | Mobile App, Desktop app, Website |
| Lin, J. ^94^ | Telemedicine | Follow up | Synchronous | FaceTime, phone call | Purpose-shifted | Health consumers & Healthcare professionals | Cardiovascular disorders | Hospital | Both | Mobile app, Telephone call |
| Ma^95^ | Telemedicine | Follow up | Asynchronous | Unspecified | Unspecified | Health consumers & Healthcare professionals | Liver illness | Hospital | Web-based | Mobile App |
| Meloni^96^ | Telemedicine | Follow up | Synchronous | Unspecified | Unspecified | Health consumers & Healthcare professionals | Diabetes | Hospital | Non-web-based | Telephone call |
| Palomba^97^ | Telemedicine | Follow up | Synchronous | Skype, WhatsApp | Purpose-shifted | Health consumers & Healthcare professionals | General | Hospital | Web-based | Mobile App, Desktop app, Website |
| Punia^98^ | Telemedicine | Follow up | Synchronous | ECO platform, FaceTime, Google Duo | Both | Health consumers & Healthcare professionals | Neurological disorders | Medical center | Both | Mobile App, Desktop app, Website, Telephone call |
| Qualliotine^99^ | Telemedicine | Follow up | Synchronous | Unspecified | Unspecified | Health consumers & Healthcare professionals | General | Hospital | Both | Mobile app, Telephone call |
| Naik100 | Telemedicine | Monitoring (patients) | Synchronous | Unspecified | Unspecified | Healthcare professionals | COVID-19 | Hospital | Web-based | Mobile app, CCTV cameras |
| Thornton^101^ | Telemedicine | Monitoring (patients) | Both | Medopad app | Built for purpose | Health consumers & Healthcare professionals | COVID-19 | Hospital | Both | Mobile app, Telephone call |
| Umoren^102^ | Telemedicine | Monitoring (patients) | Synchronous | InTouch Health | Built for purpose | Health consumers & Healthcare professionals | General | Hospital | Web-based | Mobile App, Desktop app |
| Martinez^103^ | Telemedicine | Monitoring (patients), Follow up | Both | TELEA | Built for purpose | Health consumers & Healthcare professionals | COVID-19 | Hospital | Both | Mobile app, Telephone call |
| Xu^104^ | Telemedicine | Monitoring (patients), Education, Follow up | Synchronous | eCounseling, WeChat | Both | Health consumers & Healthcare professionals | COVID-19 | Hospital | Web-based | Mobile App, Desktop app, Website |
| Vilendrer^105^ | Telemedicine | Monitoring (patients), Connecting patients and families | Synchronous | FaceTime, WebEx, Zoom, | Purpose-shifted | Health consumers & Healthcare professionals | COVID-19 | Hospital | Web-based | Mobile App, Desktop app, Website |
| Ratwani^106^ | Telemedicine | Screening | Synchronous | Unspecified | Unspecified | Health consumers & Healthcare professionals | COVID-19 | Medical center | Web-based | Mobile app, Desktop app |
| McKiever^107^ | Telemedicine | Treatment | Synchronous | Unspecified | Unspecified | Health consumers & Healthcare professionals | Prenatal and postnatal conditions | Hospital | Web-based | Mobile app, Desktop app |
| Medalia^108^ | Telemedicine | Treatment | Synchronous | WebEx, Zoom | Purpose-shifted | Health consumers & Healthcare professionals | Mental illnesses | Medical center | Web-based | Mobile App, Desktop app, Website |
| Miu^109^ | Telemedicine | Treatment | Synchronous | Unspecified | Unspecified | Health consumers & Healthcare professionals | Mental illnesses | Medical center | Both | Mobile App, Telephone call |
| Sequeira^110^ | Telemedicine | Treatment | Synchronous | Zoom | Purpose-shifted | Health consumers & Healthcare professionals | Mental illnesses | Medical center | Web-based | Mobile App, Desktop app, Website |
| Stewart^111^ | Telemedicine | Treatment | Synchronous | Vidyo | Built for purpose | Health consumers & Healthcare professionals | Mental illnesses | Medical center | Web-based | Mobile App, Desktop app |
| Sharma^112^ | Telemedicine | Treatment | Synchronous | InTouch Health, WebEx, Zoom | Both | Health consumers & Healthcare professionals | Mental illnesses | Medical center | Both | Mobile App, Desktop app, Website, Telephone call |
| Dimer^113^ | Telemedicine | Treatment, Follow up | Synchronous | Unspecified | Unspecified | Health consumers & Healthcare professionals | ENT issues | Hospital | Non-web-based | Telephone call |
| Reeves^114^ | Telemedicine, CDSS (AI-based), Dashboard, EHR, Patient portal | Accessing patient records, Booking appointments, Decision making, Diagnosing, Medical data exchange, Monitoring (patients and services), Reporting, Triage | Both | MyChart | Purpose-shifted | Health consumers & Healthcare professionals | COVID-19 | Medical center, Hospital | Both | Mobile app, Website, Telephone call, Email |
| Low^115^ | Telemedicine | Administrative support | Synchronous | Unspecified | Unspecified | Health consumers & Healthcare professionals | COVID-19 | Hospital | non-web-based | Telephone call |
| Martin^116^ | Telemedicine | Administrative support, Clinical assessment | Synchronous | HoloLens2 | Built for purpose | Healthcare professionals | COVID-19 | Hospital | Web-based | Desktop app, Headset |
| Nagaratnam^117^ | CDSS (AI-based) | Decision making | Inapplicable | e-Stroke Suite and WhatsApp | Both | Healthcare professionals | Cardiovascular disorders | Hospital | Both | Mobile App, Desktop app, Website |
| Shamout^118^ | CDSS (AI-based) | Prognosis | Inapplicable | Unspecified | Unspecified | Healthcare professionals | COVID-19 | Hospital | Non-web-based | Desktop app |
| Gong^119^ | Symptom tracker, CDSS | Monitoring (patients), Decision making | Inapplicable | Honghu Hybrid System (HHS) | Built for purpose | Decision makers | COVID-19 | Hospital | Web-based | Mobile apps |
| Miseikis^120^ | Robotic system | Personal assistant | Inapplicable | Lio | Built for purpose | Health consumers & Healthcare professionals | General | Medical center, Hospital, Community | Non-web-based | Robot |
| Rane^121^ | Robotic system | Screening | Inapplicable | Unspecified | Unspecified | Health consumers | COVID-19 | Community | Non-web-based | Robot |
| Tabaza^122^ | Robotic system | Treatment | Inapplicable | CorPath | Built for purpose | Healthcare professionals | Cardiovascular disorders | Medical center | Web-based | Robot |
| Wang, J. ^123^ | Robotic system | Diagnosis, Monitoring (patient) | Inapplicable | MGIUSA-R3 | Built for purpose | Healthcare professionals | COVID-19 | Hospital | Web-based | Robot |
| Ye^124^ | Robotic system | Diagnosis, Monitoring (patient) | Inapplicable | MGIUSA-R3 | Built for purpose | Healthcare professionals | COVID-19 | Hospital | Web-based | Robot |
| Yu^125^ | Robotic system | Diagnosis, Monitoring (patient) | Inapplicable | MGIUSA-R3 | Built for purpose | Healthcare professionals | COVID-19 | Hospital | web-based | Robot |
| Drew^126^ | Symptom tracker | Monitoring (patients) | Asynchronous | Unspecified | Unspecified | Health consumers | COVID-19 | Community | Web-based | Mobile App |
| Menni1^27^ | Symptom tracker | Monitoring (patients) | Inapplicable | Unspecified | Unspecified | Health consumers | COVID-19 | Community | Web-based | Mobile App |
| Yamamoto^128^ | Symptom tracker | Monitoring (patients) | Inapplicable | K-note | Built for purpose | Health consumers & Healthcare professionals | COVID-19 | Medical center | Web-based | Mobile App, Desktop app |
| Schinkothe^129^ | Symptom tracker, EHR | Monitoring (patients), Accessing patient records | Inapplicable | C19CC, HCP Pro App | Built for purpose | Health consumers & Healthcare professionals | COVID-19 | Medical center | Web-based | Mobile App, Website |
| Dixit^130^ | Dashboard | Monitoring (services) | Inapplicable | Unspecified | Unspecified | Decision makers | COVID-19 | Hospital | Web-based | Desktop app |
| Stevens^131^ | Dashboard | Monitoring (patients & services) | Inapplicable | Unspecified | Unspecified | Decision makers | COVID-19 & Urologic diseases | Hospital | Non-web-based | Desktop app |
| Ntshalintshali^132^ | EHR | Accessing patient records | Inapplicable | Trello | Built for purpose | Healthcare professionals | COVID-19 | Hospital | Web-based | Mobile App |
| Lai^133^ | Educational platform | Education | Inapplicable | PalliCOVID | Built for purpose | Healthcare professionals | COVID-19 | Community | Web-based | Website |
| Li, C.^134^ | Educational platform | Education | Inapplicable | Zoom | Purpose-shifted | Healthcare professionals | General | Hospital | Web-based | Mobile App, Desktop app, Website |
| Li, J.^135^ | Low-dose CT method (CareDose 4D combined with Karl 3D technology in the low dose computerized tomography) | Follow up | Inapplicable | Unspecified | Unspecified | Health consumers & Healthcare professionals | COVID-19 | Hospital | Non-web-based | Desktop app |
