# Supplementary material for: Overview of Technologies Implemented During the First Wave of the COVID-19 Pandemic: Scoping Review
Source: J Med Internet Res. 2021 Sep 14;23(9):e29136. doi: 10.2196/29136 (PMC8767979; doi:10.2196/29136)
Supplement: Multimedia Appendix 5 [file jmir_v23i9e29136_app5.docx]

# Appendix 5: Characteristics of technologies used for COVID-19 as reported in 38 studies.

| **Characteristics** | | **Study n (%)** | **Study ID** |
| --- | --- | --- | --- |
| **Technology type^1^** | Telemedicine | 23 (60.5) | [23, 26, 41, 42,49, 72, 75, 78, 82, 85, 86, 90-92, 100, 101, 103-106, 114-116] |
|  | Clinical decision support tools | 7 (18.4) | [75, 78, 85, 86, 114, 118, 119] |
|  | Symptom trackers | 6 (15.8) | [75, 119, 126-129] |
|  | Dashboards | 5 (13.2) | [75, 90, 114, 130, 131] |
|  | Robotic systems | 4 (10.5) | [121, 123-125] |
|  | Electronic health records | 4 (10.5) | [41, 114, 129, 132] |
|  | Patient portals | 1 (2.6) | [114] |
|  | Educational platforms | 1 (2.6) | [133] |
|  | Triage tools | 1 (2.6) | [90] |
|  | Low-dose CT method | 1 (2.6) | [135] |
| **Mode of telemedicine^2^** | Synchronous | 15 (65.2) | [23, 41, 42, 75, 82, 85, 86, 90, 100, 104-106, 115, 116] |
|  | Asynchronous | 2 (8.7) | [78, 91, 126] |
|  | Both | 6 (26.1) | [26, 49, 92, 101, 103, 114] |
| **Technology aim^3^** | Monitoring health status | 19 (50) | [75, 82, 90, 91, 100, 103-105, 114, 119, 123-129, 131] |
|  | Consultation | 9 (23.7) | [23, 26, 41, 42, 49, 72, 75, 78, 82] |
|  | Follow up | 7 (18.4) | [49, 72, 75, 78, 92, 103, 135] |
|  | Triage | 6 (15.2) | [49, 75, 85, 86, 90, 114] |
|  | Education | 5 (13.2) | [49, 90, 91, 104, 133] |
|  | Accessing patient records | 4 (10.5) | [41, 114, 129, 132] |
|  | Monitoring health services | 4 (10.5) | [90, 114, 130, 131] |
|  | Diagnosing | 3 (7.9) | [42, 86, 114] |
|  | Screening | 3 (7.9) | [78, 106, 121] |
|  | Administrative support | 2 (5.3) | [115, 116] |
|  | Booking appointments | 2 (5.3) | [75, 114] |
|  | Decision making | 2 (5.3) | [114, 119] |
|  | Generating reports | 2 (5.3) | [75, 114] |
|  | Connecting patients and families | 2 (5.3) | [90, 105] |
|  | Clinical assessment | 1 (2.6) | [116] |
|  | Treatment | 1 (2.6) | [82] |
|  | Medical data exchange | 1 (2.6) | [114] |
|  | Prognosis | 1 (2.6) | [118] |
| **Technology development^1,4^** | Built for purpose | 20 (83.3) | [26, 41, 42, 78, 86, 90-92, 101, 103, 104, 116, 119, 123-125, 128, 129, 132, 133] |
|  | Purpose-shifted | 5 (20.8) | [23, 72, 104, 105, 114] |
| **Social media and video-conferencing platforms^5^** | Zoom | 2 (5.3) | [72, 105] |
|  | FaceTime | 1 (2.6) | [105] |
|  | WeChat | 1 (2.6) | [104] |
|  | WebEx | 1 (2.6) | [105] |
|  | Skype | 1 (2.6) | [23] |
| **Target users** | Health consumers & Healthcare professionals | 21 (55.3) | [23, 26, 42, 49, 72, 75, 78, 85, 86, 90, 92, 101, 103-106, 114, 115, 128, 129, 135] |
|  | Healthcare professionals | 10 (2.6) | [41, 82, 100, 116, 118, 123-125, 132, 133] |
|  | Health consumers | 4 (10.5) | [91, 121, 126, 127] |
|  | Decision makers | 3 (7.9) | [119, 130, 131] |
| **Setting^6^** | Hospitals | 25 (65.8) | [26, 40, 41, 82, 85, 86, 90, 91, 100, 101, 103-105, 114-116, 118, 119, 123-125, 130-132, 135] |
|  | Medical clinics | 12 (31.8) | [23, 42, 49, 72, 78, 85, 90, 92, 106, 114, 128, 129] |
|  | Community | 5 (13.2) | [75, 121, 126, 127, 133] |
| **Internet connectivity^1^** | Web-based | 33 (86.8) | [23, 26, 41, 42, 49, 72, 75, 78, 82, 85, 86, 90-92, 100, 101, 103-106, 114, 116, 119, 123-130, 132, 133] |
|  | Non-web-based | 16 (42.1) | [23, 26, 49, 78, 85, 86, 90, 92, 101, 103, 114, 115, 118, 121, 131, 135] |
| **Venues^7^** | Mobile apps | 23 (60.5) | [23, 41, 42, 72, 75, 78, 82, 85, 90, 91, 100, 101, 103-106, 114, 119, 126-129, 132] |
|  | Desktop apps | 18 (47.4) | [23, 26, 49,72, 75, 82, 85, 86, 90, 104-106, 116, 118, 128, 130, 131, 135] |
|  | Websites | 8 (21.1) | [23, 72, 92, 104, 105, 114, 129, 133] |
|  | Telephone calls | 7 (18.4) | [26, 49, 92, 101, 103, 114, 115] |
|  | Robot | 4 (10.5) | [121, 123-125] |
|  | Emails | 3 (7.9) | [26, 92, 114] |
|  | Interphone | 1 (2.6) | [23] |
|  | Automated vital-sign monitor | 1 (2.6) | [90] |
|  | CCTV cameras | 1 (2.6) | [100] |
|  | Headset | 1 (2.6) | [116] |
| **Notes** | ^1^: Numbers do not add up as there is more than one technology in many studies.  ^2^: Number of telemedicine studies (i.e., 23) was used to calculate percentages.  ^3^: Numbers do not add up as there is more than one technology in many studies and/or many technologies were used for more than one aim.  ^4^: We were able to identify the type of technology development in 24 studies. Therefore, we used this number to calculate percentages.  ^5^: Numbers do not add up as there are more than one social media and video-conferencing platforms in many studies.  ^6^: Numbers do not add up as many technologies served patients in different settings in many studies.  ^7^: Numbers do not add up as many technologies were used through more than venues in many studies. | | |
